# Genetic characterization and molecular survey of *Babesia bovis*, *Babesia bigemina* and *Babesia ovata* in cattle, dairy cattle and yaks in China

**DOI:** 10.1186/s13071-015-1110-0

**Published:** 2015-10-09

**Authors:** Qingli Niu, Zhijie Liu, Peifa Yu, Jifei Yang, Mirza Omar Abdallah, Guiquan Guan, Guangyuan Liu, Jianxun Luo, Hong Yin

**Affiliations:** State Key Laboratory of Veterinary Etiological Biology, Key Laboratory of Veterinary Parasitology of Gansu Province, Lanzhou Veterinary Research Institute, Chinese Academy of Agricultural Science, Xujiaping 1, Lanzhou, Gansu 730046 PR China; Jiangsu Co-innovation Center for Prevention and Control of Important Animal Infectious Diseases and Zoonoses, Yangzhou, 225009 PR China

**Keywords:** *rap-1*, *ama-1*, Molecular survey, Genetic diversity, *Babesia*

## Abstract

**Background:**

Babesiosis is an important haemoparasitic disease, caused by the infection and subsequent intra-erythrocytic multiplication of protozoa of the genus *Babesia* that impacts the livestock industry and animal health. The distribution, epidemiology and genetic characterization of *B. bigemina*, *B. bovis*, and *B. ovata* in cattle in China as well as the prevalence of these protozoan agents were assessed.

**Methods:**

A total of 646 blood specimens from cattle, dairy cattle and yaks from 14 provinces were collected and tested for the presence of the three *Babesia* species via a specific nested PCR assay based on the *rap-1* and *ama-1* genes. The PCR results were confirmed by DNA sequencing. Gene sequences and the genetic characterization were determined for selected positive samples from each sampling area.

**Results:**

Of a total of 646 samples, 134 (20.7 %), 60 (9.3 %) and 10 (1.5 %) were positive for *B. bovis*, *B. bigemina* and *B. ovata* infections, respectively. Mixed infections were found in 7 of 14 provinces; 43 (6.7 %) samples were infected with *B. bovis* and *B. bigemina*. Three samples (0.5 %) exhibited a co-infection with *B. bovis* and *B. ovata*, and 6 (0.9 %) were infected with all three parasites. The *rap-1a* gene of *B. bovis* indicated a high degree of sequence heterogeneity compared with other published *rap-1a* sequences worldwide and was 85–100 % identical to *B. bovis rap-1a* sequences in Chinese isolates. *B. bigemina rap-1c* and *B. ovata ama-1* genes were nearly identical, with 97.8–99.3 % and 97.8–99.6 % sequence identity, respectively, in GenBank.

**Conclusions:**

Positive rates of *B. bovis* and *B. bigemina* infection are somewhat high in China. The *B. bovis* infection in yaks was first reported. The significant sequence heterogeneity in different variants of the *rap-1a* gene from Chinese *B. bovis* isolates might be a great threat to the cattle industry if RAP-1a protein is used as immunological antigen against *Babesia* infections in China. The data obtained in this study can be used to plan effective control strategies against babesiosis in China.

**Electronic supplementary material:**

The online version of this article (doi:10.1186/s13071-015-1110-0) contains supplementary material, which is available to authorized users.

## Background

Bovine babesiosis is a haemoparasitic disease caused by the intra-erythrocytic multiplication of apicomplexan protozoa, mainly *Babesia bovis* and *B. bigemina*, and is responsible for substantial mortality and morbidity rates and large economic losses in the livestock industry in tropical and subtropical regions. In China, the currently reported causative agents of bovine babesiosis are *B. bigemina*, *B. bovis*, *B. ovata*, *B. major*, *B. orientalis* and the recently described *Babesia* U sp. Kashi, which was derived from *Hyalomma anatolicum* collected from the field [1–5]. Among these species, *B. bovis* and *B. bigemina* are the most common species affecting bovines and are usually present in co-infections in field animals. *B. bigemina* infection is normally characterized by a low level of parasitaemia, in contrast with *B. bovis* infection, which results in high parasitaemia and a more severe disease than *B. bigemina*. Both species are distributed worldwide and transmitted by the genus *Rhipicephalus* (*Boophilus*) *microplus*, which is one of the most widely distributed and economically important ticks. These two species of *Babesia* are considered to have the greatest economic impact on cattle health. *B. major* and *B. ovata* are transmitted by *Haemaphysalis punctata* and *H. longicornis,* respectively, whereas *B. orientalis* is transmitted by *Rhipicephalus* and has thus far been determined to be highly pathogenic only to buffalo [6]. *B. divergens* has been detected in anaemic human patients but has not been isolated in cattle in China [7].

The prevalence of babesiosis correlates with the geographic distribution and activity of vector ticks. Furthermore, changing environmental conditions, especially global warming, favours tick survival and reproduction and hence a significant increase in the abundance of ticks [8].

Traditionally, thin or thick smears stained with Giemsa can detect the presence of *Babesia*, based on the morphological examination. Useful to confirm acute cases, this method has limited value for chronic cases, where only low numbers of *Babesia* exist. Moreover, the species differentiation remains challenging (refer to *B. bigemina* co-existence with *B. ovata*, with the similar morphologies between them). Serological diagnostic tests such as IFAT and ELISA have been established for a number of *Babesia* species, with the drawback of serological cross-reactions between closely related species [9]. In contrast to these methods, the application of PCR-based molecular diagnostic tools allows direct, specific and sensitive detection of parasites as well as rapid, simultaneous detection and differentiation of co-infecting *Babesia* parasites similar morphologies and with same transmitted vector ticks in a given animal [10].

The clinical signs of babesiosis are usually characterized by fever, depression, haemolytic anaemia, haemoglobinuria, and icterus, and the disease can result in death in severe cases. The presence of these signs is due to the invasion and repeated rounds of asexual multiplication of the parasites in host erythrocytes. During the invasion process, several molecules, including Associated-membrane-antigen-1 (AMA-1), Rhoptry-associated-protein-1 (RAP-1), Thrombospondin-related anonymous protein (TRAP) and spherical body proteins (SBPs), are secreted by the apical organelles and represent potential vaccine targets [11]. The highly conserved nature of the genes encoding these molecules suggests a potentially immunogenic function of these proteins.

AMA-1 protein is conserved among apicomplexan parasites [12] and has been characterized in the cattle *Babesia* species *B. bovis* [13], *B. bigemina* [14], *B. ovata* [15] and *B. divergens* [16].

In the genus *Babesia*, RAP-1 was initially described in *B. bigemina* [17] and was then characterized in all examined *Babesia* species, including *B. bovis* [18] and *B. divergens* [19]. The *rap-1* gene family in all *Babesia* species studied is characterized by the presence of multiple copies of the gene arranged in tandem head to tail. *Rap-1a* has been identified in all *Babesia* species and is an informative marker for a broader phylogenetic analysis in the genus *Babesia*. Strict sequence identity of the *rap-1b* gene copies and a single polymorphic gene copy of *rap-1c* were only found in *B. bigemina* and *B. motasi*-like group-infected sheep. *Rap-1c* is considered eligible for the characterization of field isolates of *B. bigemina* from different geographic areas [20, 21].

*Rap-1* and *ama-1* have been used as markers for the molecular diagnosis of cattle babesiosis [22–25]. The investigation of polymorphism in these genes and a comparative analysis of sequences between *Babesia* species infecting the same host in different geographic isolates may help to develop specific and sensitive molecular diagnostic tests for *Babesia* species infecting cattle around the world, and it is essential to develop effective preventive strategies before a disease outbreak. The prevalence of bovine babesiosis and the genetic diversity of these pathogens have not been well studied in China. The purpose of this study was to investigate the distribution of *B. bigemina*, *B. bovis*, and *B. ovata* in China and to determine the prevalence of these pathogens in cattle as well as to evaluate the genetic diversity of *rap-1* (*B. bovis*, *B. bigemina*) and *ama-1* (*B. ovata*), which encode potentially immunogenic proteins, among different isolates of these parasites in the world. Specific nested PCR assays were used to this end.

## Methods

### Parasites

*B. bovis* and *B. bigemina* were isolated from splenectomized cattle experimentally infected with *Rhipicephalus (Boophilus) microplus* ticks collected from Shanxian in Henan Province and Kunming in Yunnan Province, respectively, whereas *B. ovata* was isolated from *Haemaphysalis longicornis* ticks collected from pastures in Henan Province in China [4] and cryopreserved in liquid nitrogen at the Vector and Vector-borne disease (VVBD) laboratory of Lanzhou Veterinary Research Institute (LVRI), CAAS Lanzhou, China. The parasites were isolated by inoculating infected blood (10 % parasitaemia) cryopreserved in liquid nitrogen into haemoprotozoa-free splenectomized cattle. When parasitaemia was greater than 10 %, infected venous blood was collected into heparinized tubes, and the merozoites were purified for genomic DNA extraction and then used as a positive control.

### Blood sample collection

**Fig. 1:**
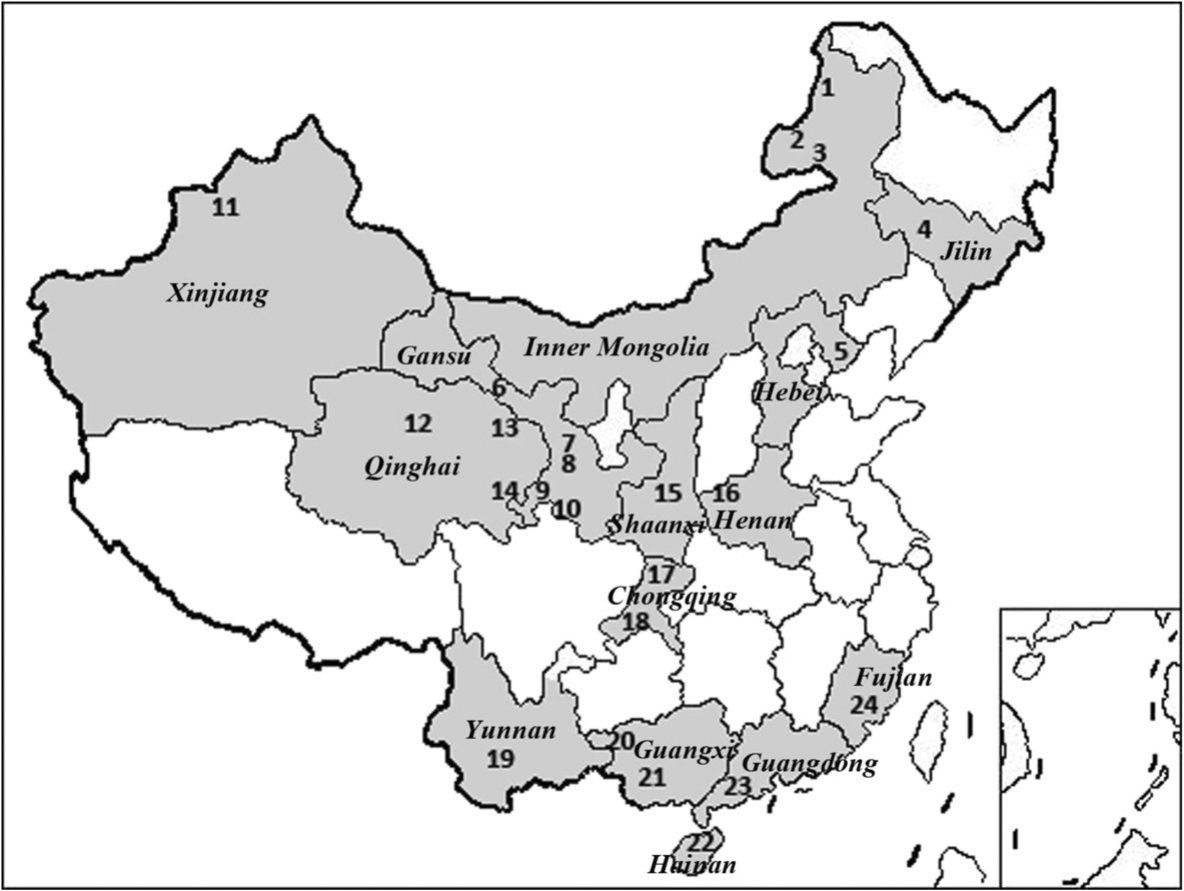
Geographical distribution of the sampling sites in China.1. Xin Barag Left Banner (cattle, *n* = 53), 2. Manzhouli City (cattle, *n* = 27), 3. Eergu’Na City (cattle, *n* = 14), 4. Changchun City (cattle, *n* = 23), 5. Tangshan City (dairy cattle, *n* = 33), 6. Lintao Country (cattle, *n* = 14), 7. Zhangye City (cattle, *n* = 14), 8. Zhuoni County of Gannan Tibetan Autonomous Prefecture (cattle, *n* = 20), 9. Lintan Country, Gannan Tibetan Autonomous Prefecture (yak, *n* = 24), 10. Luqu Country, Gannan Tibetan Autonomous Prefecture (yak, *n* = 22), 11. Yili area (cattle, *n* = 50), 12. Menyuan Country of Hui Autonomous County (yak, *n* = 14), 13. Haixing Country (cattle, *n* = 20), 14. Gangcha Country (cattle, *n* = 13), 15. Weiyuan Country (cattle, *n* = 21), 16. Shanxian Country (cattle, *n* = 51), 17. Jiangjin area (cattle, *n* = 29), 18. Wanzhou area (cattle, *n* = 24), 19. Puer City (cattle, *n* = 57), 20. Chongzuo City (cattle, *n* = 12), 21. Baise City (cattle, *n* = 20), 22. Haikou City (dairy cattle, *n* = 37), 23. Maoyuan City (dairy cattle, *n* = 29), 24. Nanping City (cattle, *n* = 25)

A total of 646 field blood samples were randomly collected from clinically healthy cattle, dairy cattle or yak from 24 different locations in 14 Chinese provinces from June 2007 to July 2014 (Fig. 1): Inner Mongolia (*n* = 94), Gansu (*n* = 94), Qinghai (*n* = 47), Xinjiang Uygur Autonomous Region (*n* = 50), Shaanxi (*n* = 21), Jilin (*n* = 23), Henan (*n* = 51), Chongqing (*n* = 53), Guangdong (*n* = 29), Guangxi (*n* = 32), Fujian (*n* = 25), Hebei (*n* = 33), Hainan (*n* = 37) and Yunnan (*n* = 57). All blood samples were collected in EDTA-coated Vacutainer tubes, transported to the laboratory with an ice pack and stored at −20 °C.

### Genomic DNA sample preparation

Genomic DNA was extracted from 300 μl of each anticoagulated blood sample and handled using a QIAamp DNA Blood Kit (QIAGEN, Maryland, USA) following the manufacturer’s instructions. Extracted DNA was eluted in 50 μl of the elution buffer and then stored at −20 °C until use. Negative control DNA was isolated from the venous blood of uninfected cattle.

### Ethical approval

This study was approved by the Animal Ethics Committee of the Lanzhou Veterinary Research Institute, Chinese Academy of Agricultural Sciences. All animals were handled in accordance with the Animal Ethics Procedures and Guidelines of the People’s Republic of China.

### Primer design

To obtain *B. bovis*, *B. bigemina* and *B. ovata rap-1* and *ama-1* gene sequences, the primer pairs of PCR and nested PCR for particular genes were designed based on sequences extracted from GenBank (under the following accession numbers: *B. bovis rap-1a*, AF030055-AF 030062, FJ588009- FJ588013; *B. bigemina rap-1c*, AY146983-AY146987, AF026272; *B. ovata ama-1*, AB634843) according to the multiple sequence alignments of each gene from *Babesia* species that infect ruminants (Table 1) for screening and sequencing. The primers targeted the *rap-1a* (*B. bovis*), *rap-1c* (*B. bigemina*), and *ama-1* (*B. ovata*) genes for amplification of the full length of or part of the homologous gene of interest. The specificity of each primer was evaluated against the DNA of various *Babesia* species.

**Table 1:** Target gene, primer combinations, amplified conditions used for PCR amplifications

Species	Target gene	PCR assay	Primer name	Sequence (5′-3′)	Tm (°C)	Product size (bp)
*B. bovis*	*rap-1a*	PCR^a^	B.borap-1aF1/ B.borap-1aR1	ACGCGAATGGTTGCGTTTCAGA	55	570
				GGCTCAGCAACATTGGCTTTCAG		
		nPCR^a^	B.borap-1aF2/ B.borap-1aR2	GGTGATTACCACTACTTCGTCAC	55	394
				CGTACGAGGTCAAGCTACCGAGCAG		
		PCR^b^	B.borap-1afull-F/B.borap-1afull-R	ATGAGAATCATTAGCGGCGTTGTCGGTT	64	1698
				TCAGAGGTATCCGGCGGTGTCTTCACCG		
		nPCR^b^	B.borap-1aseq-F/B.borap-1aseq-R	TGTACGGATGCTTTACGATTGAC	59	1358
				AGTTGAGTCGTTAGACTGAGTGGTA		
*B. bigemina*	*rap-1c*	PCR^a^	B.birap-1cF1/**B.birap-1cR** ^**c**^	AAGCAGCAGCCGTGGTACAAGCGTTGG	58	657
				**TTACGACGATCGTTTGAAGTACTTC**		
		nPCR^a^	B.birap-1cF2/**B.birap-1cR** ^**c**^	TGGCGAACTCGCAGACCAAGTAG	60	274
		PCR^b^	B.birap-1cF/**B.birap-1cR** ^**c**^	ATGATTCACTACGCTTGCCTCA	57	1530
		nPCR^b^	B.birap-1cseq-F/**B.birap-1cR** ^**c**^	TTACGCTGCTTACTACAGCTTCA	57	1054
*B. ovata*	*ama-1*	PCR^a^	B.ovama-1 F1/**B.ovama-1R1** ^**c**^	GGCAGGTGCCTGCGTGGCGATCG	62	600
				**GAGCAACAACAGCGCCGTAGTAATCACG**		
		nPCR^a^	B.ovama-1 F2/ B.ovama-1R2	TGATATCGATATCGACCTTGATTC	62	310
				GAGCTGTCACCATTGTCCTTAACAC		
		PCR^b^	B.ovama-1seqF/**B.ovama-1R1** ^**c**^	GATACGAGGCTGTCGGTAGC	62	1234
		nPCR^b^	B.ovama-1seqF/B.ovama-1R2		58	1041

^a^For screening analysis

^b^For sequencing analysis

^c^bold data: The sequences of these primeres appeared twice or more times

### Detection of field samples with two sets PCR assays

The specificity of both primer sets was initially tested to amplify the targeted gene of interest, and other parasitic pathogens were used as controls. All of the DNA field samples were then amplified to screen for the presence of parasites. The two sets of primers were used to amplify the shorter fragment of each gene (Table 1). The PCR amplification was conducted in a final volume of 25 μl, composed of 12.5 μl of Premix Taq™ (TaKaRa Taq™ Version 2.0 plus dye), 0.5 μl (20 pmol) of each external primer, 3 μl of the template DNA sample, 8.5 μl of double-distilled water and 1 μl of positive control DNA. The amplification parameters for PCR were an initial denaturation at 94 °C for 5 min followed by 35 cycles of denaturation at 94 °C for 30 s, annealing at 55 °C or 58 °C or 62 °C for 45 s, and extension at 72 °C for 1 min, with a final extension at 72 °C for 10 min. Each product from the first PCR reaction (1 μl) was then added to the second PCR mixture, and Ex Taq Hot-Start DNA polymerase (TaKaRa) was used to amplify the internal fragment using the internal primers.

The expected size of the amplicons was analysed on 1.5 % agarose gels with GoldView I nucleotide stains (Solarbio) and visualized under UV illumination.

For the sequencing of obtained positive samples for the study of the phylogenetic analysis among different isolates, both sets of primers were used to amplify full-length or longer sequences of each gene from randomly selected positive samples. PCR mixtures and amplification were performed as described above using the appropriate annealing temperatures. The amplicons (approximately 1.4 kb for *B. bovis rap-1a* and approximately 1 kb for *B. bigemina rap-1c* and *B. ovata ama-1*) from secondary PCR were used for phylogenetic analysis.

### Cloning and sequencing

The PCR products from selected field-positive samples (11 samples for *B. bovis rap-1a*, 10 samples for *B. bigemina rap-1c* and 3 samples for *B. ovata ama-1*) were purified using a MiniBEST DNA Fragment Purification Kit (TaKaRa) and cloned into pGEM®-T Easy Vector Systems (Promega) and then transformed into Trans5α Chemically Competent Cells (TransGen Biotech) according to the manufacturers’ instructions. Colonies were selected by direct colony PCR using vector primers. Multiple sub-colonies from each sample were selected, cultured in 3 ml LB broth and sequenced. The long inserts were sequenced by successively designing internal primers.

### Bioinformatics analysis

All sequences obtained in this study were subjected to a blast search on the NCBI website (http://blast.ncbi.nlm.nih.gov/Blast.cgi) using the BLASTn program.

Multiple sequence alignments were analysed using the ClustalW 2.0.12 software ClustalW: Multiple alignment. Phylogenetic analysis was performed using the MEGA 6.06 software [26]. The three different genes were analysed using the “neighbour-joining” method based on genetic distances. Phylogenetic trees were constructed from other isolates of *B. bovis*, *B. bigemina* and *B. ovata* by including selected gene sequences and related database sequences in GenBank to show relationships between different isolates from our study and other regions in the world.

### Statistical analysis

The 95 % confidence intervals (95 % CIs) for the overall prevalence values of each *Babesia* species were calculated using IBM SPSS Statistics version 19.0.

## Results

### PCR detection of three Babesia species from field samples

All primers amplified only their respective target sequence for individual *Babesia* species; no cross reaction was observed. No amplification was observed when cattle genomic DNA and water were used as controls (data not shown). Field blood samples extracted from a total of 646 cattle, dairy cattle or yak from 14 Chinese provinces were detected with both nPCR assays. The first round of nested PCR assays screening primers specific for the *rap-1* or *ama-1* genes for the presence of the three *Babesia* species of interest were developed to detect *B. bovis*, *B. bigemina* and *B. ovata* in cattle samples. The samples were amplified with a length of ~394 bp for *B. bovis*, ~277 bp for *B. bigemina* and ~310 bp for *B. ovata* for the genes of interest, respectively. The results of the nested PCR amplification for positive sample screenings are summarized in Table 2. In summary, cattle blood samples infected with *B. bovis* were found in 12 of 14 surveyed provinces, and 8 provinces of 14 exhibited *B. bigemina* infections. Only cattle from Henan Province showed *B. ovata* infection. *Babesia* DNA was found in 134 (*B. bovis*), 60 (*B. bigemina*), and 10 (*B. ovata*) of the 646 cattle, dairy cattle or yak from all regions. The total infection rates indicated by nPCR were 20.7 % for *B. bovis*, 9.3 % for *B. bigemina* and 1.5 % for *B. ovata*. The total *B. bovis* infection rate in yaks was 13 % (8/60), with 27 % (6/22) in Luqu Country and 8.3 % (2/24) in Lintan Country in Gansu province, respectively. No positive sample was found in yaks collected from Qinghai province. Of all these positive samples, 43 had double infections of *B. bovis* and *B. bigemina* in seven provinces; three and six samples had *B. bovis* + *B. ovata* double infections and *B. bovis + B. bigemina + B. ovata* infections, respectively; and triple infections in which all *Babesia* species were detected occurred only in samples from Henan Province. The highest prevalence of *B. bovis* and *B. bigemina* infections occurred in Fujian and Hainan Provinces, respectively.

**Table 2:** Detection results of *B. bovis*, *B. bigemina* and *B. ovata* in field blood samples

Province	Date	No. of samples	Positive rate (%)			Mix Positive rate (%)			
	Year/month		*B. bovis*	*B. bigemina*	*B. ovata*	*B. bo* + *B. bi*	*B. bi* + *B. ov*	*B. bo* + *B. ov*	*B. bo* + *B. bi* + *B. ov*
Inner Mongolia	2013.6	94	1 (1)	1 (1)	0	0	0	0	0
Gansu	2013.5	94	22 (23.4)	0	0	0	0	0	0
Qinghai	2013.1	47	3 (6.4)	0	0	0	0	0	0
Xinjiang	2014.7	50	0	0	0	0	0	0	0
Shaanxi	2013.7	21	0	0	0	0	0	0	0
Jilin	2008.6	23	1 (4.3)	0	0	0	0	0	0
Henan	2007.5	51	28 (54.9)	13 (25.5)	10 (19.6)	11 (21.6)	0	3 (5.9)	6 (11.8)
Chongqing	2011.5	53	13 (24.5)	3 (5.7)	0	2 (3.8)	0	0	0
Guangdong	2011.6	29	1 (3.4)	0	0	0	0	0	0
Guangxi	2011.4	32	12 (37.5)	2 (6.3)	0	2 (6.3)	0	0	0
Fujian	2012.5	25	15 (60)	6 (24)	0	5 (20)	0	0	0
Hebei	2008.4	33	1 (3)	1 (3)	0	(1 (3)	0	0	0
Hainan	2013.5	37	16 (43.2)	23 (62.2)	0	15 (40.5)	0	0	0
Yunnan	2014.7	57	21 (36.8)	11 (19.3)	0	7 (12.3)	0	0	0
Total		646	134 (20.7)	60 (9.3)	10 (1.5)	43 (6.7)	0	3 (0.5)	6 (0.9)

*B. bo*: *B. bovis*

*B. bi*: *B. bigemina*

*B. ov*: *B. ovata*

### Sequence variation of rap-1 and ama-1 genes in the Chinese B. bovis, B. bigemina and B. ovata isolates

The amplification of the *rap-1* and *ama-1* genes using the appropriate primers (B.borap-1aseq-F/R; B.birap-1cseq-F/B.birap-1cR and B.ovama-1seqF/B.ovama-1R2, Table 1) produced three different amplicons of 1358, 1057 and 1041 bp, respectively. The size of the amplicons might correspond to the expected length of *rap-1* or *ama-1*. Ten clones per sample containing inserts were sequenced for *B. bovis*, *B. bigemina* and *B. ovata*, all of them exhibiting homologous *rap-1* or *ama-1* genes in the databases. A representative sequence from each sample was selected based on the sequence alignments of ten clones. Sequencing and sequence analysis confirmed that the nPCR assays were specific for the target genes and *Babesia* species.

#### B. bovis rap-1a

Sequence alignment of *rap-1a* genes from 11 Chinese strains demonstrated 99 nucleotide substitutions and 36 amino acid modifications (non-synonymous) that were affected by 42 of these nucleotide substitutions. Among these substitutions, 58 nucleotide substitutions were found in Henan strains 1 and 3 (Additional file 1a). The common molecular features of the RAP-1 protein in all studied *Babesia* spp. indicated the presence of patches of conserved motifs in the first N-terminal of approximately 300 amino acids. In this study, the amplified region (463 aa) began at aa 103 and included an N-terminal conserved region from aa 1 to aa 211 compared with the full-length RAP-1a sequence of *B. bovis* of 565 aa). Seven modifications between aa 1 and aa 211 (N-terminal) and 10 modifications between aa 396 and aa 463 were identified. The differences (20 aa modifications) in the protein sequences were mostly limited to a specific region composed of repeated sequences (23 aa per repeat) between aa 212 and aa 395 (Additional file 1b). In the *rap-1a* genes, these regions were localized at the C-terminal of the gene, starting between 634 and 1185 bp in the sequences amplified in this study, with a total length of 552 bp (Additional file 1a).

Thirteen single substitutions at the first and 13 at the second positions were synonymous (26/36), but only four substitutions at the third position were synonymous (4/36), whereas double substitutions at the first and third or second and third positions were synonymous (6/36) (Additional file 1b).

#### B. bigemina rap-1c

Precise sequences of the *rap-1c* derived from 10 strains demonstrated 16 substitutions at different positions (Additional file 2). Six amino acid modifications affected seven codons (non-synonymous) by nucleotide substitutions among the isolates. Four amino acid modifications (nucleotide positions: 602, 608, 749 + 751 and 1024) occurred in two Yunnan isolates. Two substitutions (602, 608) at the first position, two substitutions at the first and third positions (749 + 751) and one substitution (1024) at the third position, as well as one aa change (nucleotide position: 889, substitution at the second position), occurred in the Chongqing isolate. Another aa modification (nucleotide position: 969, substitution at the third position) occurred in the HAN2 isolate (Additional file 2). Nine substitutions were synonymous.

The per cent identity among the Chinese isolates of *B. bovis* and *B. bigemina* based on the *rap-1a* and *rap-1c* genes was determined and is shown in Table 3.

**Table 3:**
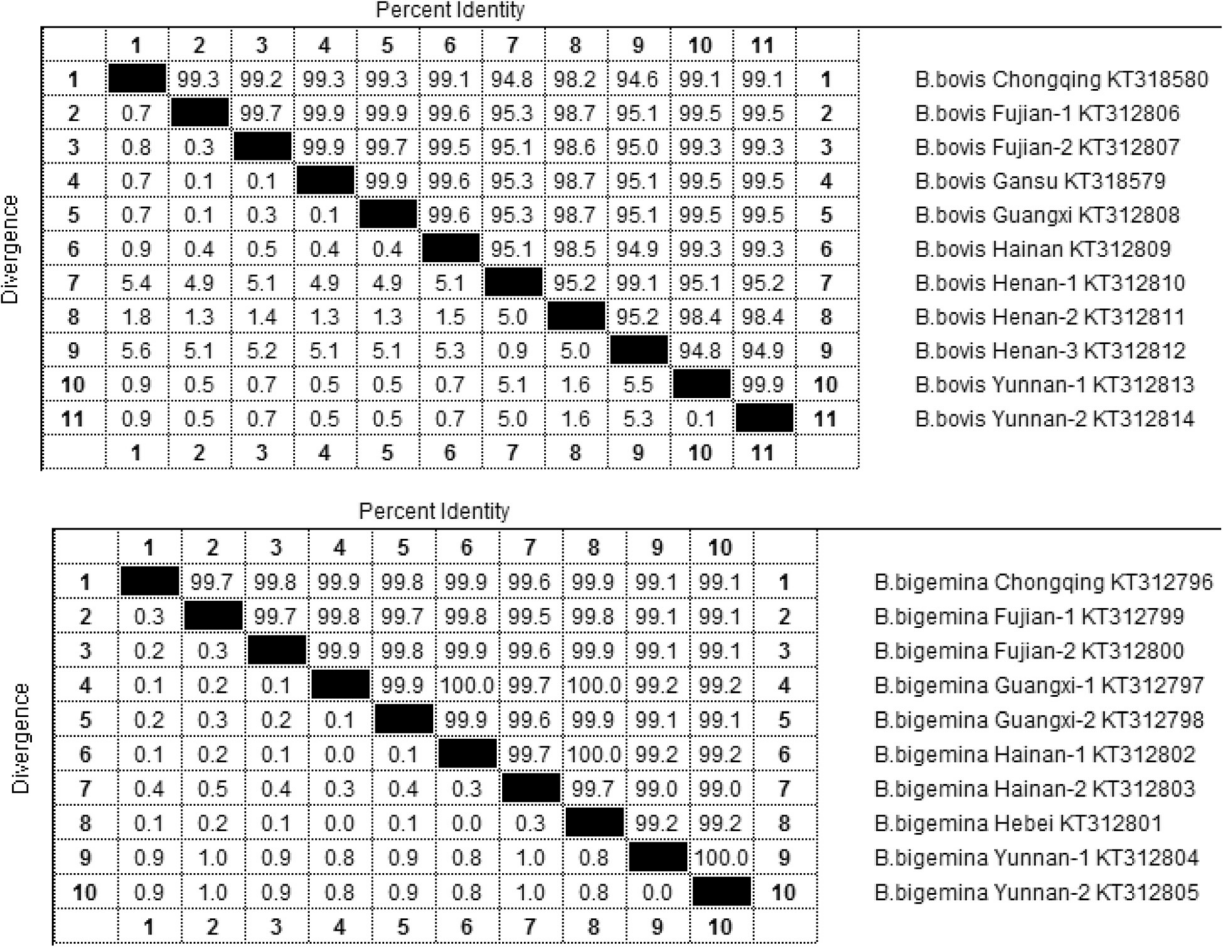
Percent identity of *B. bovis rap-1a* and *B. bigemina rap-1c* gene sequences of Chinese isolates deduced after CLUSTAL W alignment

#### B. ovata ama-1

The sequences of the *ama-1* gene from three isolates of the Henan strains were obtained and revealed 100 % identity between isolates 1 and 2, whereas 12 nucleotide substitutions involving a 1 aa modification were found in isolate 3 compared with the other two isolates.

### Phylogenetic analysis

Three phylogenetic trees were constructed based on *B. bovis rap-1a* (*n* = 11)*, B. bigemina rap-1c* (*n* = 10) and *B. ovata ama-1* (*n* = 3) gene sequences determined in the present study by the neighbour-joining method using the software MEGA6.06. These gene sequences were deposited in GenBank in various countries.

#### B. bovis rap-1a

**Fig. 2:**
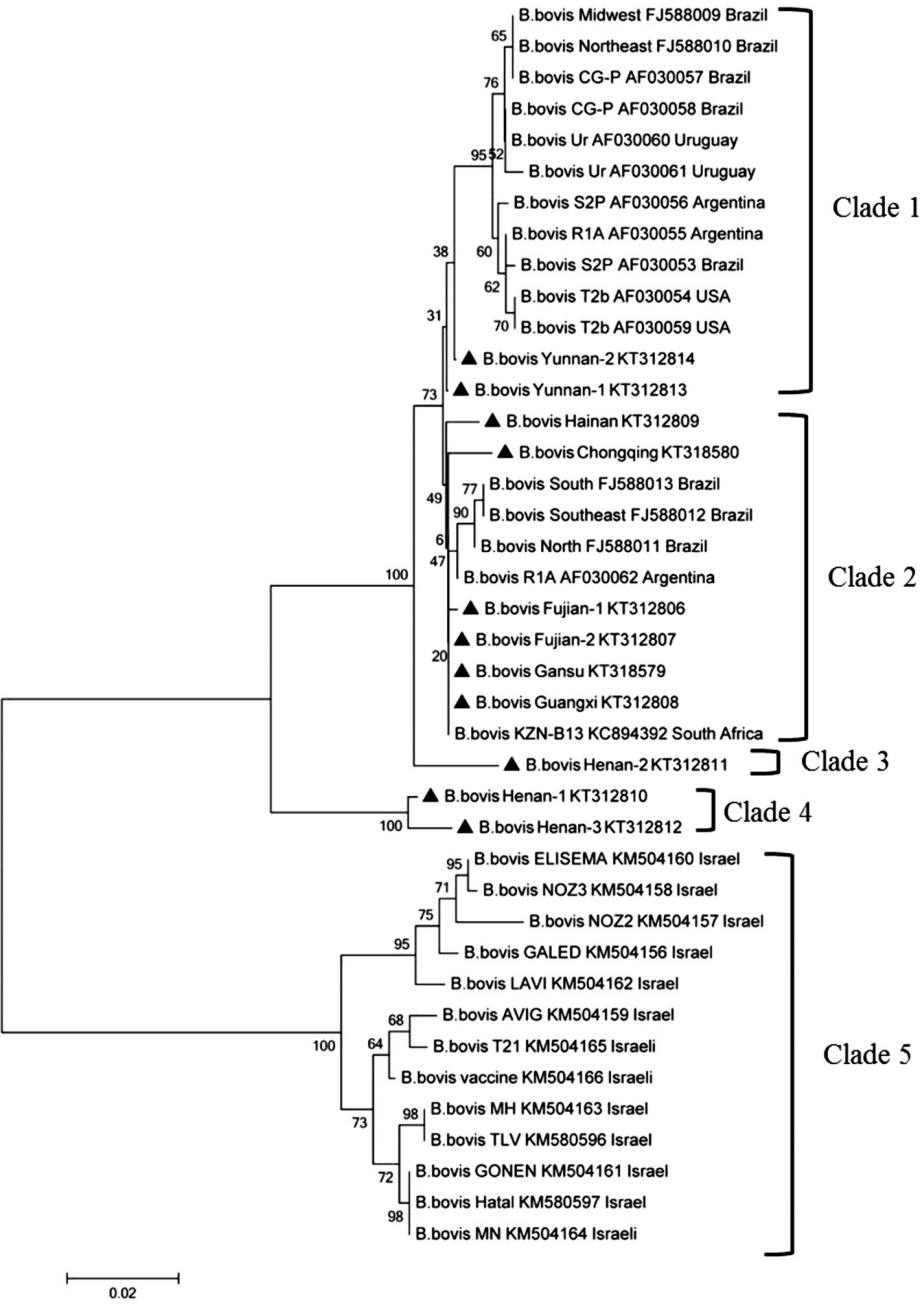
Phylogenetic tree of the nucleotidic sequences of *B. bovis rap-1a* and all sequences of this gene deposited in GenBank from different countries, the accession numbers and countries are shown after isolate name. The *rap-1a* sequences obtained in this study were indicated with bold triangle. The tree was inferred using the neighbor joining method of MEGA6.06, bootstrap values are shown at each branch point. Numbers above the branch demonstrate bootstrap support from 1000 replications. All sites of the alignment containing insertions-deletions, missing data were eliminated from the analysis (option “complete deletion”)

The *rap-1* phylogenetic tree indicated that the *rap-1a* sequences from our study formed five clades that were generally distinct from the *rap-1a* sequences from other countries, with several sub-branches (Fig. 2). Two Yunnan isolates formed a sub-branch in the first clade, forming a sister clade with the *B. bovis rap-1a* sequences from the USA, Argentina, Brazil and Uruguay. The isolates from Fujian, Gansu, and Guangxi Provinces formed the second clade, clustered with several sub-branches. The Guangxi isolate was more closely related to *B. bovis* isolate KZN-B13, which is from South Africa, whereas isolates from Chongqing and Hainan were also in this clade but formed separate, single branches. The *rap-1a* sequence (Henan-2) from Henan cattle formed a single clade (clade 3), whereas two other Henan isolates formed a different single clade (clade 4). The previously published *rap-1a* sequences of *B. bovis* of cattle from Israel formed a separate clade (clade 5). In general, the results show the presence of high heterogeneity among the *rap-1* sequences of different isolates of *B. bovis* species worldwide (Fig. 2).

#### B. bigemina rap-1c

**Fig. 3:**
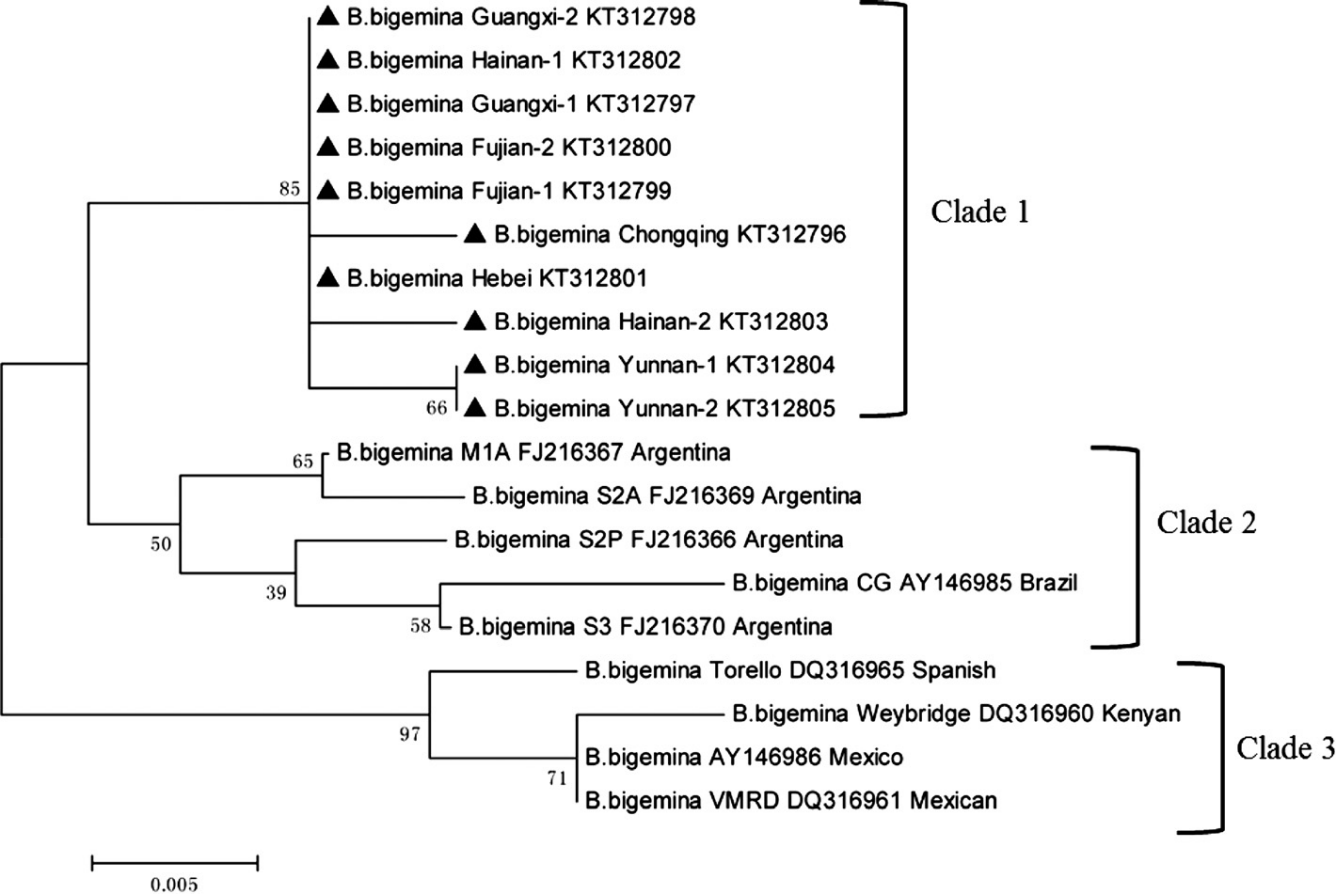
Phylogenetic tree of the nucleotidic sequences of *B. bigemina rap-1c* and all sequences of this gene deposited in GenBank from different countries, the accession numbers and countries are shown after isolate name. The *rap-1c* sequences obtained in this study were indicated with bold triangle. The tree was inferred using the neighbor joining method of MEGA6.06, bootstrap values are shown at each branch point. Numbers above the branch demonstrate bootstrap support from 1000 replications. All sites of the alignment containing insertions-deletions, missing data were eliminated from the analysis (option “complete deletion”)

Three distinct clades were present (Fig. 3) in the sequences of the *B. bigemina rap-1c* phylogenetic tree. All of the *rap-1c* sequences of *B. bigemina* from ten Chinese isolates were within the first clade with five sister branches: the first branch comprised Fujian, Guangxi and Hainan-1 *rap-1c*; the fifth comprised two Yunnan isolates; and the other three branches contained only one isolate. The previously published *rap-1c* sequences of *B. bigemina* from Argentinian and Brazilian cattle formed clade 2, whereas the *rap-1c* sequences from Spanish, Kenyan and Mexican cattle formed clade 3 (Fig. 3).

#### B. ovata ama-1

**Fig. 4:**
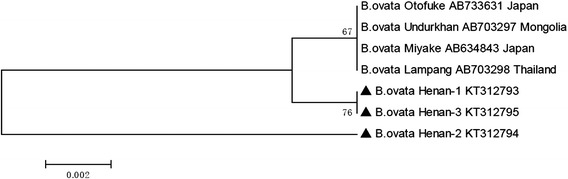
Phylogenetic tree of the nucleotidic sequences of *B. ovata ama-1* and all sequences of this gene deposited in GenBank from different countries, the accession numbers and countries are shown after isolate name. The *ama-1* sequences obtained in this study were indicated with bold triangle. The tree was inferred using the neighbor joining method of MEGA6.06, bootstrap values are shown at each branch point. Numbers above the branch demonstrate bootstrap support from 1000 replications. All sites of the alignment containing insertions-deletions, missing data were eliminated from the analysis (option “complete deletion”)

A limited number of *B. ovata ama-1* sequences were found in GenBank. A phylogenetic tree was constructed based on all *B. ovata ama-1* sequences deposited in GenBank, and three sequences obtained in this study were used (Fig. 4). All the *ama-1* sequences from *B. ovata* formed two main clades. Two Henan isolates clustered in a sister branch with the *B. ovata ama-1* from Japan, Mongolia and Thailand, forming the first main clade, and another *B. ovata* Henan-2 isolate formed a separate clade (Fig. 4).

### Nucleotide accession numbers

All sequences, including 11 for *B. bovis rap-1a*, 10 for *B. bigemina rap-1c* and 3 for *B. ovata ama-1* genes, were deposited in GenBank with the following accession numbers: 9 (KT312806- KT312814) and 2 (KT318579-KT318580) sequences of *B. bovis rap-1a* from seven provinces (two from Fujian, one from Guangxi, one from Hainan, three from Henan, two from Yunnan, one from Gansu and one from Chongqing), 10 (KT312796**-**KT312805) sequences of *B. bigemina rap-1c* from six provinces (one from Chongqing, two from Guangxi, two from Fujian, one from Hebei, two from Hainan, and two from Yunnan) and 3 (KT312793-KT312795) sequences of *B. ovata ama-1* from only Henan Province.

## Discussion

In China, babesiosis is caused by various protozoan parasites of the genus *Babesia*. Many of these agents are highly pathogenic for ruminant cattle, buffalo, and yak [2, 27]. Cattle grazing areas are distributed in a broad range in China. Ruminant babesiosis, which is mainly caused by the pathogenic agents *B. bovis* and *B. bigemina,* is considered one of the most prevalent tick-borne diseases and has an important economic impact in China. Four *B. ovata* isolates (Lushi, Ningxian, Zhangjiachuan and Wenchuan) from Henan, Guansu and Sichuan Provinces have been reported in China [4]. However, the distribution of this pathogen may be much wider, depending on the distribution of its transmitted vector *H. longicornis* [28].

Previous studies reported the prevalence of cattle *Babesia* species in field blood collected from different Chinese sites, and the results showed that the distribution of the *Babesia* species varied considerably in the different areas. This variability may have been caused by the distribution of the different tick vectors from different sampling sites [29, 30].

In our study, the prevalence of three cattle babesial pathogens was investigated in a broad geographic range in the field. A total of 646 bovine blood samples were collected from 14 provinces located in northeast, northwest, central and southern China and were analysed using nested PCR assays. The findings revealed that *B. bovis* was the most widespread bovine haemoprotozoan species in Chinese cattle, but *B. bigemina* was less widespread, similar to the results of a previous study of these parasites. However, the positive rate of *Babesia* parasites was apparently higher than in previous findings using multiplex PCR and LAMP assays based on the gene target ITS [27, 28].

It is worth mentioning that few reports have described the *B. bigemina* infection in yaks from in Xinjiang Uygur Autonomous Region, northwestern China and Tianzhu Tibetan Autonomous County (TTAC), Gansu Province, northwest China and Nepal [31–33], however, the infection detection was only based on the serological survey, the identification at species level needs to be further confirmed. The recent report confirmed the *B. bigemina* infection in yaks in India using a sensitive PCR based on the small-subunit rRNA (SS rRNA) gene of *B. bigemina* and by further sequence analysis as well as by studying its restriction profile of the amplified fragment [34]. No positive samples about *B. bigemina* infection in yaks were found in our study. For the infection and prevalence of *B. bovis* in yaks, no scientific survey has been conducted so far. In the present study, the detected yaks with *B. bovis* positive were from Gannan Tibetan Autonomous Prefecture, Luqu and Lintan Countries, located at east and northeast edges of the Qinghai Tibet Plateau, Gansu Province of China, respectively. The weather is cold, damp and with low oxygen content, altitude of 2900–4200 m and 2200–3900 m, they have hypoxia tolerance and cold-resistant ability in the environment. Detection and molecular confirmation of *B. bovis* infection in yaks was first reported. The studies of importance for the tick vector, *R.* (*B.*) *microplus* transmitted *Babesia* to yaks is limited. Recent study indicated that the *R. (B.) microplus* could be collected from field at high altitude of 1800–2200 m [35]. Saravanan *et al.* [34] reported the yaks are moved to higher altitude of more than 4500 m during summer and brought back during winter. Due to animal migration, the *R.* (*B.*) *microplus* are likely to transfer to those regions with higher altitudes. However, the presence and survival of *R. (B.) microplus* at Luqu and Lintan Countries with higher altitudes is unknown. Therefore the potential appearance of babesiosis of *R. (B.) microplus* in yaks needs to be further studied. In addition, the sharing of common grazing lands with cattle in survey areas results in common vector born *B. bovis* infections in yaks might be another explanation.

The widespread co-existence (7 provinces) of *B. bovis* and *B. bigemina* implies the presence and distribution of the same tick vectors (*R.* (*B.*) *microplus*) in the surveyed areas. *R.* (*B.*) *microplus* is one of the most important tick species causing babesiosis in cattle and is widespread in China. Approximately 24 provinces have reported the occurrence of this tick species [36].

*B. ovata* infection was found in only 10 samples from Henan Province, which is unusual because this parasite is reportedly widespread in China [4]. In addition, the tick vector of *B. ovata* is *H. longicornis,* which is also a prevalent tick species and is widely distributed in Jilin, Hebei, Gansu, and Shaanxi Provinces and in Chongqing city [36]. The lower infection rate of *B. ovata* in our study is thus most likely a result of sampling different regions, depending on the marker used for the molecular detection or the lower sensitivity of the PCR method used for *Babesia* species.

The overall infection rates of *B. bovis*, *B. bigemina* and *B. ovata* were 20.7 % (95 % CI = 10.8–32.6), 9.3 % (95 % CI = 2.8–20.6) and 1.5 % (95 % CI = 0–4.2), respectively, in blood samples using the nested PCR assay (Table 2). These rates are comparable to other studies using multiplex PCR and LAMP assays; positive rates for *B. bovis* and *B. bigemina* were 7.7 and 5.8 %, respectively, with multiplex PCR [30] and 13.5 and 7.3 %, respectively, with LAMP [29]. Therefore, the wide prevalence of bovine blood samples positive for *B. bovis* and *B. bigemina*, as well as co-infections of two parasites in this study, was not surprising. The results suggest that the nested PCR assay developed based on the molecular markers *rap-1* and *ama-1* in our study can be considered as a potential diagnostic method for epidemiological investigations.

Functional protein secretion during the parasite’s asexual growth cycle in the blood stage might be an effective control strategy for babesiosis [37]. The *ama-1* and *rap-1* gene products are considered potential antigen candidates because of their role in red blood cell invasion [38, 39]. Understanding the genetic characteristics and evolution of these genes is essential for designing a protective antigen against babesiosis.

In *B. bovis*, conservation of the gene sequence among geographically distinct isolates of *rap-1a* would be advantageous for the development of a diagnostic antigen in China, and immune control strategies against *B. bovis* in China could be designed in light of the genetic diversity of this parasite. In this study, we therefore investigated the sequence polymorphism of these genes from randomly selected positive samples from distinct geographical provinces and compared these with genes from other countries worldwide. Although *rap-1* is a multiple-gene family, only the *rap-1a* gene type was described in *B. bovis* [18]. The molecular features of the *rap-1* gene family show that the N-terminal region of the RAP-1 sequence is more highly conserved among babesial parasites than the C-terminal region, and several sequences encode conserved or degenerate repeats located at the C-terminal in many *Babesia* species, including *B. bovis* [40].

In our study, the amplification range for the *B. bovis rap-1a* gene was focused on the fragment starting from 305 bp of the full-length (1358 bp) gene to the 3′ end. The sequence analysis demonstrated sequence diversity, but some conserved features of the *rap-1* family were also evident: a conserved 14 amino acid motif (PLTLPNPYQLDAAF) and several shorter conserved oligopeptide motifs (YKTYL) close to the N-terminal region (Additional file 1b). Manual alignment revealed 8 degenerate repeats in a variable region of the C-terminal, consisting of 69 nt per repeat that corresponded to the published *B. bovis* RAP-1a sequence (GenBank accession number: ACM4406). Analysis of these repeat sequences in our study revealed 20 aa differences among 11 different isolates (Additional file 1b). The results suggest that targeting a common region to produce a protective antigen from the C-terminal region of protein might be ineffective because of the great genetic variation in *rap-1a* among different isolates, even though these repeats were found to contain the predominant predicted linear protein B-epitope in the RAP-1a sequences. In general, these repeats are thought to mediate functions involved in parasite survival and immune evasion [41]. The phylogenetic analysis of *rap-1a* sequences from different worldwide isolates also indicated genetic variation; 11 *B. bovis* Chinese isolates were segregated into three separate clades (Fig. 2). In contrast, a similar previous study showed the *rap-1a* sequences of distinct isolates from the same country were located on the same clade of the tree and reported a high degree of conservation of sequences at the nucleotide and amino acid levels among isolates [22, 24]. The characterization of the genetic diversity of this region in *B. bovis rap-1a* Chinese isolates could be used as an informative marker for a broader phylogenetic analysis of the genus *Babesia*.

The ClustalW alignment of multiple *rap-1a* sequences of all *B. bovis* Chinese isolates shows that the identity among *B. bovis* isolates is greater than 99 %, except for the Henan isolates. The per cent identity of three Henan isolates compared to the other eight *B. bovis* isolates was 94.6–95.2 % (Table 3); the *rap-1a* sequences of Henan isolates showed a higher degree of heterogeneity. A comparison of the *rap-1* sequences of Chinese isolates with published *rap-1a* sequences from South Africa, Argentina, USA, Brazil and Israel revealed identities ranging from 85–100 % [22, 42–44].

From many perspectives, sequence analysis of the *rap-1* sequences obtained (1358 bp) revealed less sequence heterogeneity at the 5′ end of the *rap-1* gene and greater sequence heterogeneity in the middle of the repeated region and at the 3′ end of the *rap-1* gene in Chinese isolates, as well as in isolates elsewhere in the world. These findings suggest a lack of conservation of the C-terminal repeat region and indicate that B-cell-rich epitopes could be used as a species-specific diagnostic ELISA antigen for the detection of antibodies to *B. bovis*.

The presence of only one *rap-1c* gene copy located at the 3′ end of the *rap-1* locus was found only in the *B. bigemina* genome in cattle, and *rap-1c* has proven to be transcribed but not translated [39] and could thus be used as specific molecular marker [20]. In our study, the genetic polymorphism of the *rap-1c* gene from *B. bigemina* isolates from China and other countries was analysed. The sequences show limited polymorphism among different Chinese isolates, with a maximum of 6 amino acid substitutions (Additional file 2). The per cent identity of 10 *B. bigemina* Chinese isolates was high, ranging from 99 to 100 % (Guangxi isolate with Hainna-1and Hebei and two Yunnan isolates) (Table 3). The phylogenetic analysis indicated that the *rap-1c* sequences of the *B. bigemina* isolates deduced in the present study were clustered together in several branches. Furthermore, a comparison of the *rap-1c* in Chinese *B. bigemina* field isolates with sequences derived from Brazilian and Mexican *B. bigemina* isolates revealed that the total sequence identity among the isolates ranged from 97.8 to 99.3 % [39].

Our findings agree with previous studies that demonstrated that the nucleotide alignment of *rap-1c* from field isolates had an overall identity ranging from 99.0 to 100 %, with the largest number of changes in the amino acid sequences in the C-terminal of RAP-1c [39, 44].

*B. ovata* is considered less pathogenic to cattle than *B. bovis* and *B. bigemina*, and the similar morphologies and 18S RNA gene sequences of *B. ovata* and *B. bigemina* make it difficult to distinguish these two parasites [15]. Further studies are necessary to identify an available marker and to develop a molecular detection assay to differentiate *B. ovata* from *B. bigemina*.

The *ama-1* genes are highly conserved among apicomplexan parasites and have been characterized in several species of *Babesia* [12]. Several recent studies have used a diagnostic PCR assay based on the *ama-1* gene to identify and specifically detect *B. ovata* from questing ticks and cattle [45–47]. The *ama-1* gene sequences share an overall identity ranging from 97.8 to 99.6 % at the nucleotide level in a comparison of Chinese isolates with those from other countries [47]. To our knowledge, this is the first report on *B. ovata* infection in China. *B. ovata ama-1* sequences from Henan Province indicated high conservation in three Henan isolates. In contrast to the *B. bovis* and *B. bigemina* parasites in the samples tested, a low prevalence of *B. ovata* in only one province of 14 was observed. A similar result was obtained in a previous study. *B. ovata* infection exhibits lower parasitaemia in infected cattle than *B. bovis* and *B. bigemina,* thus, it is possible that a low concentration of *B. ovata* DNA exists in field samples that could not be detected by PCR [47]. This possibility suggests that additional molecular markers and an adequate number of samples from individual locations of more provinces should be tested.

## Conclusions

We have successfully developed an appropriate and specific nested PCR approach for the detection and discrimination of the important *Babesia* parasites from different geographical locations in China as well as for epidemiological investigations. *B. bovis* and *B. bigemina* were highly prevalent in the sampling areas of the 14 provinces surveyed, but a low presence of *B. ovata* parasites was observed. Further studies concerning the prevalence of *B. ovata* should be performed to confirm the presence of this parasite in China. Specific serodiagnosis and/or molecular detection based on the *rap-1a* gene of *B. bovis* in China will allow for epidemiological surveys.

Moreover, it is the first report on detection and molecular confirmation of *B. bovis* infection in yaks. Taken together, the data presented in this study suggest that the high sequence heterogeneity in the *rap-1a* gene from Chinese *B. bovis* isolates might be a great threat to the cattle industry, if RAP-1a protein is used as immunological antigen against *Babesia* infections in China.

## Additional files

Additional file 1:
**a.** **Alignment of partial nucleotide sequences of** ***rap-1a*** **gene from different Chinese isolates.** FJ2: *B. bovis rap-1a* isolate Fujian 2; GS: *B. bovis rap-1a* isolate Guansu; FJ1: *B. bovis rap-1a* isolate Fujian 1;GX: *B. bovis rap-1a* isolate Guangxi; HAN: *B. bovis rap-1a* isolate Hainan; YN1: *B. bovis rap-1a* isolate Yunnan 1; YN2: *B. bovis rap-1a* isolate Yunnan 2; CQ: *B. bovis rap-1a* isolate Chongqing; HEN2: *B. bovis rap-1a* isolate Henan 2; HEN 1: *B. bovis rap-1a* isolate Henan 1; HEN3: *B. bovis rap-1a* isolate Henan3. Nucleotide substitutions indicated with yellow background. Among these substitutions, nucleotide substitutions affected amino acid modifications indicated with red background. **b**. Alignment of partial amino acid sequences of *rap-1a* gene from different Chinese isolates. FJ2: *B. bovis* RAP-1a isolate Fujian 2; GS: *B. bovis* RAP-1a isolate Guansu; GX: *B. bovis* RAP-1a isolate Guangxi; CQ: *B. bovis* RAP-1a isolate Chongqing; HAN: *B. bovis* RAP-1a isolate Hainan; YN1: *B. bovis* RAP-1a isolate Yunnan 1; FJ1: *B. bovis* RAP-1a isolate Fujian 1; YN2: *B. bovis* RAP-1a isolate Yunnan 2; HEN2: *B. bovis* RAP-1a isolate Henan 2; HEN 1: *B. bovis* RAP-1a isolate Henan 1; HEN3: *B. bovis* RAP-1a isolate Henan3. Repeats in RAP-1a gene from different isolates are underlined. (DOC 1086 kb) Additional file 2:
**Variability of** ***B. bigemina rap-1c*** **gene.** Positions that differ between the different isolates of sequences obtained from gene amplification and cloning for each isolate. (DOC 46 kb)
