# Supplementary material for: Genetic characterization and molecular survey of Babesia bovis, Babesia bigemina and Babesia ovata in cattle, dairy cattle and yaks in China
Source: Parasit Vectors. 2015 Oct 9;8:518. doi: 10.1186/s13071-015-1110-0 (PMC4600270; doi:10.1186/s13071-015-1110-0)
Supplement: Additional file 1: — a. Alignment of partial nucleotide sequences of rap-1a gene from different Chinese isolates. FJ2: B. bovis rap-1a isolate Fujian 2; GS: B. bovis rap-1a isolate Guansu; FJ1: B. bovis rap-1a isolate Fujian 1;GX: B. bovis rap-1a isolate Guangxi; HAN: B. bovis rap-1a isolate Hainan; YN1: B. bovis rap-1a isolate Yunnan 1; YN2: B. bovis rap-1a isolate Yunnan 2; CQ: B. bovis rap-1a isolate Chongqing; HEN2: B. bovis rap-1a isolate Henan 2; HEN 1: B. bovis rap-1a isolate Henan 1; HEN3: B. bovis rap-1a isolate Henan3. Nucleotide substitutions indicated with yellow background. Among these substitutions, nucleotide substitutions affected amino acid modifications indicated with red background. b. Alignment of partial amino acid sequences of rap-1a gene from different Chinese isolates. FJ2: B. bovis RAP-1a isolate Fujian 2; GS: B. bovis RAP-1a isolate Guansu; GX: B. bovis RAP-1a isolate Guangxi; CQ: B. bovis RAP-1a isolate Chongqing; HAN: B. bovis RAP-1a isolate Hainan; YN1: B. bovis RAP-1a isolate Yunnan 1; FJ1: B. bovis RAP-1a isolate Fujian 1; YN2: B. bovis RAP-1a isolate Yunnan 2; HEN2: B. bovis RAP-1a isolate Henan 2; HEN 1: B. bovis RAP-1a isolate Henan 1; HEN3: B. bovis RAP-1a isolate Henan3. Repeats in RAP-1a gene from different isolates are underlined. (DOC 1086 kb) [file 13071_2015_1110_MOESM1_ESM.doc]

FJ2 TGTACGGATGCTTTACGATTGACAATGTCAAATATCCGTTGTATCAAGAGTACCAACCTC 60

GS TGTACGGATGCTTTACGATTGACAATGTCAAATATCCGTTGTATCAAGAGTACCAACCTC 60

FJ1 TGTACGGATGCTTTACGATTGACAATGTCAAATATCCGTTGTATCAAGAGTACCAACCTC 60

GX TGTACGGATGCTTTACGATTGACAATGTCAAATATCCGTTGTATCAAGAGTACCAACCTC 60

HAN TGTACGGATGCTTTACGATTGACAATGTCAAATATCCGTTGTATCAAGAGTACCAACCTC 60

YN1 TGTACGGATGCTTTACGATTGACAATGTCAAATATCCGTTGTATCAAGAGTACCAACCTC 60

YN2 TGTACGGATGCTTTACGATTGACAATGTCAAATATCCGTTGTATCAAGAGTACCAACCTC 60

CQ TGTACGGATGCTTTACGATTGACAATGTCAAATATCCGTTGTATCAAGAGTACCAACCTC 60

HEN2 TGTACGGATGCTTTACGATTGACAATGACAAATATCCGTTGTATCAAGAGTACCAACCTC 60

HEN1 TGTACGGATGCTTTACGATTGACAATGTCAAATATCCGTTGTATCAAGAGTACCAACCTC 60

HEN3 TGTACGGATGCTTTACGATTGACAATGTCAAATATCCGTTGTATCAAGAGTACCAACCTC 60

*************************** ********************************

FJ2 TATCTCTTCCAAACCCTTACCAGTTGGATGCTGCGTTCAGATTGTTCAAAGAGAGTGCAT 120

GS TATCTCTTCCAAACCCTTACCAGTTGGATGCTGCGTTCAGATTGTTCAAAGAGAGTGCAT 120

FJ1 TATCTCTTCCAAACCCTTACCAGTTGGATGCTGCGTTCAGATTGTTCAAAGAGAGTGCAT 120

GX TATCTCTTCCAAACCCTTACCAGTTGGATGCTGCGTTCAGATTGTTCAAAGAGAGTGCAT 120

HAN TATCTCTTCCAAACCCTTACCAGTTGGATGCTGCGTTCAGATTGTTCAAAGAGAGTGCAT 120

YN1 TATCTCTTCCAAACCCTTACCAGTTGGATGCTGCGTTCAGATTGTTCAAAGAGAGTGCAT 120

YN2 TATCTCTTCCAAACCCTTACCAGTTGGATGCTGCGTTCAGATTGTTCAAAGAGAGTGCAT 120

CQ TGTCTCTTCCAAACCCTTACCAGTTGGATGCTGCTTTCAGATTGTTCAAAGAGAGTGCAT 120

HEN2 TATCTCTTCCAAACCCTTACCAGTTGGATGCTGCGTTCAGATTGTTCAAAGAGAGTGCAT 120

HEN1 TATCTCTTCCAAACCCTTACCAGTTGGATGCTGCGTTCAGATTGTTCAAAGAGAGTGCAT 120

HEN3 TATCTCTTCCAAACCCTTACCAGTTGGATGCTGCGTTCAGATTGTTCAAAGAGAGTGCAT 120

* ******************************** *************************

FJ2 CCAATCCTGCTAAGAACAGCGTAAAACGCGAATGGTTGCGTTTCAGAAATGGAGCGAACC 180

GS CCAATCCTGCTAAGAACAGCGTAAAACGCGAATGGTTGCGTTTCAGAAATGGAGCGAACC 180

FJ1 CCAATCCTGCTAAGAACAGCGTAAAACGCGAATGGTTGCGTTTCAGAAATGGAGCGAACC 180

GX CCAATCCTGCTAAGAACAGCGTAAAACGCGAATGGTTGCGTTTCAGAAATGGAGCGAACC 180

HAN CCAATCCTGCTAAGAACAGCGTAAAACGCGAATGGTTGCGTTTCAGAAATGGAGCGAACC 180

YN1 CGAACCCTGCCAAGTACAGCGTAAAACGCGAATGGTTGCGTTTCAGAAATGGAGCGAACC 180

YN2 CGAACCCTGCCAAGAACAGCGTAAAACGCGAATGGTTGCGTTTCAGAAATGGAGCGAACC 180

CQ CGAATCCTGCTAAGAACAGCGTAAAACGCGAATGGTTGCGTTTCAGAAATGGAGCGAACC 180

HEN2 CCAATCCTGCTAAGAACAGCGTAAAACGCGAATGGTTGCGTTTCAGAAATGGAGCGAACC 180

HEN1 CCAATCCTGCTAAGAACAGCGTAAAACGCGAATGGTTGCGTTTCAGAAATGGAGCGAACC 180

HEN3 CCAATCCTGCTAAGAACAGCGTAAAACGCGAATGGTTGCGTTTCAGAAATGGAGCGAACC 180

* ** ***** *** *********************************************

FJ2 ATGGTGATTACCACTACTTCGTCACTGGTCTGTTGAACAACAATGTTTTGCACGAGGAAG 240

GS ATGGTGATTACCACTACTTCGTCACTGGTCTGTTGAACAACAATGTTTTGCACGAGGAAG 240

FJ1 ATGGTGATTACCACTACTTCGTCACTGGTCTGTTGAACAACAATGTTGTGCACGAGGAAG 240

GX ATGGTGATTACCACTACTTCGTCACTGGTCTGTTGAACAACAATGTTGTGCACGAGGAAG 240

HAN ATGGTGATTACCACTACTTCGTCACTGGTCTGTTGAACAACAATGTTGTGCACGAGGAAG 240

YN1 ATGGTGATTACCACTACTTCGTCACTGGTCTGTTGAACAACAATGTTGTGCACGAGGAAG 240

YN2 ATGGTGATTACCACTACTTCGTCACTGGTCTGTTGAACAACAATGTTGTGCACGAGGAAG 240

CQ ATGGTGATTACCACTACTTCGTCACTGGTCTGTTGAACAACAATGTTGTGCACGAGGAAG 240

HEN2 ATGGTGATTACCACTACTTCGTCACTGGTCTGTTGAACAACAATGTTGTGCACGAGGAAG 240

HEN1 ATGGTGATTACCACTACTTCGTCACTGGTCTGTTGAACAACAATGTTGTGCACGAGGAAG 240

HEN3 ATGGTGATTACCACTACTTCGTCACTGGTCTGTTGAACAGCAATGTTGTGCACGAGGAAG 240

*************************************** ******* ************

FJ2 GAACTACCGATGTTGAATATCTTGTCAACAAGGTACTCTATATGGCTACCATGAACTACA 300

GS GAACTACCGATGTTGAATATCTTGTCAACAAGGTACTCTATATGGCTACCATGAACTACA 300

FJ1 GAACTACCGATGTTGAATATCTTGTCAACAAGGTACTCTATATGGCTACCATGAACTACA 300

GX GAACAACCGATGTTGAATATCTTGTCAACAAGGTACTCTATATGGCTACCATGAACTACA 300

HAN GAACTACCGATGTTGAATATCTTGTCAACAAGGTACTCTATATGGCTACCATGAACTACA 300

YN1 GAACTACCGATGTTGAATATCTTGTCAACAAGGTACTCTATATGGCTACCATGAACTACA 300

YN2 GAACTACCGATGTTGAATATCTTGTCAACAAGGTACTCTATATGGCTACCATGAACTACA 300

CQ GAACTACCGATGTTGAATATCTTGTCAACAAGGTACTCTATATGGCTACCATGAACTACA 300

HEN2 GAACTACCGATGTTGAATATCTTGTCAACAAGGTACTCTATATGGCTACCATGAACTACA 300

HEN1 GAACTACCGATGTTGAATATCTTGTCAACAAGGTACTCTATATGGCTACCATGAACTACA 300

HEN3 GAACTACCGATGTTGAATATCTTGTCAACAAGGTACTCTATATGGCTACCATGAACTACA 300

**** *******************************************************

FJ2 AGACTTATTTGACAGTAAACAGTATGAACGCCAAGTTTTTCAACAGATTCAGCTTCACTA 360

GS AGACTTATTTGACAGTAAACAGTATGAACGCCAAGTTTTTCAACAGATTCAGCTTCACTA 360

FJ1 AGACTTATTTGACAGTAAACAGTATGAACGCCAAGTTTTTCAACAGATTCAGCTTCACTA 360

GX AGACTTATTTGACAGTAAACAGTATGAACGCCAAGTTTTTCAACAGATTCAGCTTCACTA 360

HAN AGACTTATTTGACAGTAAACAGTATGAACGCCAAGTTTTTCAACAGATTCAGCTTCACTA 360

YN1 AGACTTATTTGACAGTAAACAGTATGAACGCCAAGTTCTTCAACAGATTCAGCTTCACTA 360

YN2 AGACTTATTTGACAGTAAACAGTATGAACGCCAAGTTCTTCAACAGATTCAGCTTCACTA 360

CQ AGACTTATTTGACAGTAAACAGTATGAACGCCAAGTTTTTCAACAGATTCAGCTTCACTA 360

HEN2 AGACTTATTTGACAGTAAACAGTATGAACGCCAAGTTTTTCAACAGATTCAGCTTCACTA 360

HEN1 AGACTTATTTGACAGTAAACAGTATGAACGCCAAGTTCTTCAACAGGTTCAGCTTCACTA 360

HEN3 AGACTTATTTGACAGTAAACAGTATGAACGCCAAGTTTTCCAACAGATTCAGCTTCACTA 360

************************************* * ****** *************

FJ2 CAAAGATATTCAGCCGTCGTATTAGGCAAACATTGAGTGATATCATCAGGTGGAACGTTC 420

GS CAAAGATATTCAGCCGTCGTATTAGGCAAACATTGAGTGATATCATCAGGTGGAATGTTC 420

FJ1 CAAAGATATTCAGCCGTCGTATTAGGCAAACATTGAGTGATATCATCAGGTGGAATGTTC 420

GX CAAAGATATTCAGCCGTCGTATTAGGCAAACATTGAGTGATATCATCAGGTGGAATGTTC 420

HAN CAAAGATATTCAGCCGTCGTATTAGGCAAACATTGAGTGATATCATCAGGTGGAATGTTC 420

YN1 CAAAGATATTCAGCCGTCGTATTAGGCAAACATTGAGTGATATCATCAGGTGGAATGTTC 420

YN2 CAAAGATATTCAGCCGTCGTATTAGGCAAACATTGAGTGATATCATCAGGTGGAATGTTC 420

CQ CAAAGATATTCAGCCGTCGTATTAGGCAAACATTGAGTGATATCATCAGGTGGAATGTTC 420

HEN2 CAAAGATATTCAGCCGTCGTATTAGGCAAACATTGAGTGATATCATCAGGTGGAATGTTC 420

HEN1 CAAAGATATTCAGTCGTCGTATTAGGCAAACATTGAGTGATATCATCAGGTGGAATGTTC 420

HEN3 CAAAGATATTCAGCCGTCGTATTAGGCAAACATTGAGTGATATCATCAGGTGGAATGTTC 420

************* ***************************************** ****

FJ2 CTGAAGATTTTGAAGAAAGGAGCATCGAACGTATCACTCAACTTACTAGCAGCTACGAAG 480

GS CTGAAGATTTTGAAGAAAGGAGCATCGAACGTATCACTCAACTTACTAGCAGCTACGAAG 480

FJ1 CTGAAGATTTTGAAGAAAGGAGCATCGAACGTATCACTCAACTTACTAGCAGCTACGAAG 480

GX CTGAAGATTTTGAAGAAAGGAGCATCGAACGTATCACTCAACTTACTAGCAGCTACGAAG 480

HAN CTGAAGATTTTGAAGAAAGGAGCATCGAACGTATCACTCAACTTACTAGCAGCTACGAAG 480

YN1 CTGAAGATTTTGAAGAAAGGAGCATCGAACGTATCACTCAACTTACTAGCAGCTACGAAG 480

YN2 CTGAAGATTTTGAAGAAAGGAGCATCGAACGTATCACTCAACTTACTAGCAGCTACGAAG 480

CQ CTGAAGATTTTGAAGAAAGGAGCATCGAACGTATCACTCAACTTACTAGCAGCTACGAAG 480

HEN2 CTGAAGATTTTGAAGAAAGGAGCATCGAACGTATCACTCAACTTACTAGCAGCTACGAGG 480

HEN1 CTGAAGATTTTGAAGAAAGGAGCATCGAACGTATCACTCAACTTACTAGCAGCTACGAGG 480

HEN3 CTGAAGATTTTGAAGAAAGGAGCATCGAACGTATCACTCAACTTACTAGCAGCTACGAGG 480

********************************************************** *

FJ2 ATTACATGTTGACCCAGATTCCAACTCTTTCCAAGTTTGCACGTCGTTATGCTGACATGG 540

GS ATTACATGTTGACCCAGATTCCAACTCTTTCCAAGTTTGCACGTCGTTATGCTGACATGG 540

FJ1 ATTACATGTTGACCCAGATTCCAACTCTTTCCAAGTTTGCACGTCGTTATGCTGACATGG 540

GX ATTACATGTTGACCCAGATTCCAACTCTTTCCAAGTTTGCACGTCGTTATGCTGACATGG 540

HAN ATTACATGTTGACCCAGATTCCAACTCTTTCCAAGTTTGCACGTCGTTATGCTGACATGG 540

YN1 ATTACATGTTGACCCAGATTCCAACTCTTTCCAAGTTTGCACGTCGTTATGCTGACATGG 540

YN2 ATTACATGTTGACCCAGATTCCAACTCTTTCCAAGTTTGCACGTCGTTATGCTGACATGG 540

CQ ATTACATGTTGACCCAGATTCCAACTCTTTCCAAGTTTGCACGTCGTTATGCTGACATGG 540

HEN2 ATTACATGCTGACCCAGGTCCCAACTCTTTCCAAGTTTGCACGTCGTTATGCTGACATGG 540

HEN1 ATTACATGCTGACCCAGGTCCCAACTCTTTCCAAGTTTGCACGTCGTTATGCTGACATGG 540

HEN3 ATTACATGCTGACCCAGGTCCCAACTCTTTCCAAGTTTGCACGTCGTTATGCTGACATGG 540

******** ******** * ****************************************

FJ2 TGAAGAAGGTTCTGCTCGGTAGCTTGACCTCGTACGTTGAAGCTCCTTGGTACAAAAGAT 600

GS TGAAGAAGGTTCTGCTCGGTAGCTTGACCTCGTACGTTGAAGCTCCTTGGTACAAAAGAT 600

FJ1 TGAAGAAGGTTCTGCTCGGTAGCTTGACCTCGTACGTTGAAGCTCCTTGGTACAAAAGAT 600

GX TGAAGAAGGTTCTGCTCGGTAGCTTGACCTCGTACGTTGAAGCTCCTTGGTACAAAAGAT 600

HAN TGAAGAAGGTTCTGCTCGGTAGCTTGACCTCGTACGTTGAAGCTCCTTGGTACAAAAGAT 600

YN1 TGAAGAAGGTTCTGCTCGGTAGCTTGACCTCGTACGTTGAAGCTCCTTGGTACAAAAGAT 600

YN2 TGAAGAAGGTTCTGCTCGGTAGCTTGACCTCGTACGTTGAAGCTCCTTGGTACAAAAGAT 600

CQ TGAAGAAGGTTCTGCTCGGTAGCTTGACCTCGTACGTTGAAGCTCCTTGGTACAAAAGAT 600

HEN2 TTAAGAAGGTTCTGCTCGGTAGCTTGACCTCGTACGTTGAAGCTCCTTGGTACAAAAGAT 600

HEN1 TTAAGAAGGTTCTGCTCGGTAGCTTGACCTCGTACGTTGAAGCTCCTTGGTACAAAAAAT 600

HEN3 TTAAGAAGGTTCTGCTCGGTAGCTTGACCTCGTACGTTGAAGCTCCTTGGTACAAAAAAT 600

* ******************************************************* **

FJ2 GGATAAAGAAATTCAGAGACTTTTTCTCTAAAAACGTTACCCAACCTACAAAGAAGTTCA 660

GS GGATAAAGAAATTCAGAGACTTTTTCTCTAAAAACGTTACCCAACCTACAAAGAAGTTCA 660

FJ1 GGATAAAGAAATTCAGAGACTTTTTCTCTAAAAACGTTACCCAACCTACAAAGAAGTTCA 660

GX GGATAAAGAAATTCAGAGACTTTTTCTCTAAAAACGTTACCCAACCTACAAAGAAGTTCA 660

HAN GGATAAAGAAATTCAGAGACTTTTTCTCTAAAAACGTTACCCAACCTACAAAGAAGTTCA 660

YN1 GGATAAAGAAATTCAGAGACTTTTTCTCTAAAAACGTTACCCAACCTACAAAGAAGTTCA 660

YN2 GGATAAAGAAATTCAGAGACTTTTTCTCTAAAAACGTTACCCAACCTACAAAGAAGTTCA 660

CQ GGATAAAGAAATTCAGAGACTTTTTCTCTAAAAACGTTACCCAACCTACAAAGAAGTTCA 660

HEN2 GGATAAAGAAATTCAGAGACTTTTTCTCTAAAAACGTTACCCAACCTACAAAGAAGTTCA 660

HEN1 GGATAAAGAAATTCAGAGACTTTTTCTCTAAAAACGTTACCCAACCTACAAAGAAGTTCA 660

HEN3 GGATAAAGAAATTCAGAGACTTTTTCTCTAAAAACGTTACCCAACCTACAAAGAAGTTCA 660

************************************************************

FJ2 TCGAGGATACTAACGAAGTTACCAAAAACTATCTGAAAGCCAATGTTGCTGAGCCCACTA 720

GS TCGAGGATACTAACGAAGTTACCAAAAACTATCTGAAAGCCAATGTTGCTGAGCCCACTA 720

FJ1 TCGAGGATACTAACGAAGTTACCAAAAACTATCTGAAAGCCAATGTTGCTGAGCCCACTA 720

GX TCGAGGATACTAACGAAGTTACCAAAAACTATCTGAAAGCCAATGTTGCTGAGCCCACTA 720

HAN TCGAGGATACTAACGAAGTTACCAAAAACTATCTGAAAGCCAATGTTGCTGAGCCCACTA 720

YN1 TCGAGGATACTAACGAAGTTACCAAAAACTATCTGAAAGCCAATGTTGCTGAGCCCACTA 720

YN2 TCGAGGATACTAACGAAGTTACCAAAAACTATCTGAAAGCCAATGTTGCTGAGCCCACTA 720

CQ TCGAGGATACTAACGAAGTTACCAAAAACTATCTGAAAGCTAATGTTGCTGAGCCCACTA 720

HEN2 TCGAGGATACTAACGAAGTTACCAAAAACTATCTGAAAGCCAATGTTGCTGAGCCCACTA 720

HEN1 TCGAGGAGACTAACGAGGTGACCAAAAACTATCTCAAAACCAATGTTTCTGAGCCCACCA 720

HEN3 TCGAGGAGACTAACGAGGTGACCAAAAACTATCTCAAAACCAATGTTTCTGAGCCCACCA 720

******* ******** ** ************** *** * ****** ********** *

FJ2 AAAAGTTTATGCAGGACACTCACGAAAAAACCAAAGGCTATCTGAAAGAGAATGTAGCCG 780

GS AAAAGTTTATGCAGGACACTCACGAAAAAACCAAAGGCTATCTGAAAGAGAATGTAGCCG 780

FJ1 AAAAGTTTATGCAGGACACTCACGAAAAAACCAAAGGCTATCTGAAAGAGAATGTAGCCG 780

GX AAAAGTTTATGCAGGACACTCACGAAAAAACCAAAGGCTATCTGAAAGAGAATGTAGCCG 780

HAN AAAAGTTTATGCAGGACACTCACGAAAAAACCAAAGGCTATCTGAAAGAGAATGTAGCCG 780

YN1 AAAAGTTTATGCAGGACACTCACGAAAAAACCAAAGGCTATCTGAAAGAGAATGTAGCCG 780

YN2 AAAAGTTTATGCAGGACACTCACGAAAAAACCAAAGGCTATCTGAAAGAGAATGTAGCCG 780

CQ AAAAGTTTATGCAGGACACTCACGAAAAAACCAAAGGCTATCTGAAAGAGAATGTAGCCG 780

HEN2 AAAAGTTTATGCAGGACACTCACGAAAAAACCAAAGGCTATCTGAAAGAGAATGTAGCCG 780

HEN1 AAAAGTTCATGCAGGACACTCACGAAAAAACCAAAGGCTATCTGAAAGAGAATGTAGCCG 780

HEN3 AAAAGTTCATGCAGGACACTCACGAAAAAACCAAAGGCTATCTGAAAGAGAATGTAGCCG 780

******* ****************************************************

FJ2 AACCTACTAAGACTTTTTTCAAGGAGGCTCCTCAAGTCACCAAACACTTCTTCGATGAGA 840

GS AACCTACTAAGACTTTTTTCAAGGAGGCTCCTCAAGTCACCAAACACTTCTTCGATGAGA 840

FJ1 AACCTACTAAGACTTTTTTCAAGGAGGCTCCTCAAGTCACCAAACACTTCTTCGATGAGA 840

GX AACCTACTAAGACTTTTTTCAAGGAGGCTCCTCAAGTCACCAAACACTTCTTCGATGAGA 840

HAN AACCTACTAAGACTTTTTTCAAGGAGGCTCCTCAAGTCACCAAACACTTCTTCGATGAGA 840

YN1 AACCTACTAAGACTTTTTTCAAGGAGGCTCCTCAAGTCACCAAACACTTCTTCGATGAGA 840

YN2 AACCTACTAAGACTTTTTTCAAGGAGGCTCCTCAAGTCACCAAACACTTCTTCGATGAGA 840

CQ AACCTACTAAGACTTTTTTCAAGGAGGCTCCTCAAGTCACCAAACACTCCTTCGATGAGA 840

HEN2 AACCTACTAAGACTTTTTTCAAGGAGGCTCCCCAAGCCACTAAGCACTTCTTAGACGAGA 840

HEN1 AACCTACTAAGACCTTTTTCAAGGAGGCTCCTCAAGTCACCAAACACTTCTTAGACGAGA 840

HEN3 AACCTACTAAGACCTTTTTCAAGGAGGCCCCCCAAGTCACCAGACACTTCTTAGACGAGA 840

************* ************** ** **** *** * **** *** ** ****

FJ2 ACATTGGCCAACCCACCAAGGAGTTTTTCAGGGAAGCTCCCCAAGCCACTAAACATTTCC 900

GS ACATTGGCCAACCCACCAAGGAGTTTTTCAGGGAAGCTCCCCAAGCCACTAAACATTTCC 900

FJ1 ACATTGGCCAACCCACCAAGGAGTTTTTCAGGGAAGCTCCCCAAGCCACTAAACATTTCC 900

GX ACATTGGCCAACCCACCAAGGAGTTTTTCAGGGAAGCTCCCCAAGCCACTAAACATTTCC 900

HAN ACATTGGCCAACCCACCAAGGAGTTTTTCAGGGAAGCTCCCCAAGCCACTAAGCACTTCT 900

YN1 ACATTGGCCAACCCACCAAGGAGTTTTTCAGGGAAGCTCCCCAAGCCACTAAACATTTCC 900

YN2 ACATTGGCCAACCCACCAAGGAGTTTTTCAGGGAAGCTCCCCAAGCCACTAAACATTTCC 900

CQ ACATTGGCCAACCCACCAAGGAGTTTTTCAGGGAAGCTCCCCAAGCCACTAAACATTTCC 900

HEN2 ATATTGCTCAACCCACCAAGGAGTTTTTCAGGGAAGCTCCCCGAGCCACTAAACATTTCC 900

HEN1 ACATTGGCCAACCCACCAAAGAGTTTTTCAGGGAAGCCCCCCAAGCCACTAAACATTTCC 900

HEN3 ACATTGGCCAACCCACCAAAGAGTTTTTCAAGGAGGCCCCCCAAGCCACTAAACATTTCC 900

* **** *********** ********** *** ** **** ********* ** ***

FJ2 TAGACGAAAACATCGGTCAACCAACCAAGGAGTTCTTCAGGGAGGCTCCCCAAGCCACTA 960

GS TAGACGAAAACATCGGTCAACCAACCAAGGAGTTCTTCAGGGAGGCTCCCCAAGCCACTA 960

FJ1 TAGACGAAAACATCGGTCAACCAACCAAGGAGTTCTTCAGGGAGGCTCCCCAAGCCACTA 960

GX TAGACGAAAACATCGGTCAACCAACCAAGGAGTTCTTCAGGGAGGCTCCCCAAGCCACTA 960

HAN TAGACGAAAACATCGGTCAACCAACCAAGGAGTTCTTCAGGGAGGCTCCCCAAGCCACTA 960

YN1 TAGACGAAAACATCGGTCAACCAACCAAGGAGTTCTTCAGGGAGGCTCCCCAAGCCACTA 960

YN2 TAGACGAAAACATCGGTCAACCAACCAAGGAGTTCTTCAGGGAGGCTCCCCAAGCCACTA 960

CQ TAGACGAAAACATCGGTCAACCAACCAAGGAGTTTTTCAGGGAGGCTCCCCAAGCAACTA 960

HEN2 TAGACGAAAACATCGGTCAACCAACCAAGGAGTTCTTCAGGGAGGCTCCCCAAGCCACTA 960

HEN1 TAGACGAAAACATCGGTCAACCAACCAAGGAGTTCTTTAGGGAAGCTCCTCAAGCCACCA 960

HEN3 TAGACGAAAACATCGGTCAACCAACCAAGGAGTTCTTTAGGGAAGCTCCTCAAGCCACCA 960

********************************** ** ***** ***** ***** ** *

FJ2 AGCACTTCTTAGACGAGAATATTGCTCAACCTACTAAAGAATTTTTCAGGGATGTCCCTC 1020

GS AGCACTTCTTAGACGAGAATATTGCTCAACCTACTAAAGAATTTTTCAGGGATGTCCCTC 1020

FJ1 AGCACTTCTTAGACGAGAATATTGCTCAACCTACTAAAGAATTTTTCAGGGATGTCCCTC 1020

GX AGCACTTCTTAGACGAGAATATTGCTCAACCTACTAAAGAATTTTTCAGGGATGTCCCTC 1020

HAN AGCACTTCTTAGACGAGAATATTGCTCAACCTACTAAAGAATTTTTCAGGGATGTCCCTC 1020

YN1 AGCACTTCTTAGACGAGAATATTGCTCAACCTACTAAAGAATTTTTCAGGGATGTCCCTC 1020

YN2 AGCACTTCTTAGACGAGAATATTGCTCAACCTACTAAAGAATTTTTCAGGGATGTCCCTC 1020

CQ AGCACTTCTTAGACGAGAATATTGCTCAACCTACTAAAGAATTTTTCAGGGATGTCCCTC 1020

HEN2 AGCACTTCTTAGACGAGAATATTGCTCAACCTACTAAAGAATTTTTCAGGGATGTCCCTC 1020

HEN1 AGCACTTCTTAGACGAGAATATTGCTCAACCTACTAAAGAATTTTTCAGAGATGTCCCTC 1020

HEN3 AGCACTTCTTAGACGAGAATATTGCTCAACCTACTAAAGAATTTTTCAGAGATGTCCCTC 1020

************************************************* **********

FJ2 AAGTCACCAAGAAGGTTATAACTGAGAACATTGCTCAACCAACTAAGGAATTCCTTAAGG 1080

GS AAGTCACCAAGAAGGTTATAACTGAGAACATTGCTCAACCAACTAAGGAATTCCTTAAGG 1080

FJ1 AAGTCACCAAGAAGGTTATAACTGAGAACATTGCTCAACCAACTAAGGAATTCCTTAAGG 1080

GX AAGTCACCAAGAAGGTTATAACTGAGAACATTGCTCAACCAACTAAGGAATTCCTTAAGG 1080

HAN AAGTCACCAAGAAGGTTATAACTGAGGACATTGCTCAACCAACTAAGGAATTCCTTAAGG 1080

YN1 AAGTCACCAAGAAGGTTATAACTGAGAACATTGCTCAACCAACTAAGGAGTTCCTTAAGG 1080

YN2 AAGTCACCAAGAAGGTTATAACTGAGAACATTGCTCAACCAACTAAGGAGTTCCTTAGGG 1080

CQ AAGTCACCAAGAAGGTTATAACTGAGAACATTGCTCAACCAACTAAGGAATTCCTTAAGG 1080

HEN2 AAGTCACCAAGAAGGTTATAACTGAGAACATTGCTCAACCAACTAAGGAATTCCTTAAGG 1080

HEN1 AAGTCACCAAGAAGGTTATAAGTGAGAACATTGCTCAACCAACTAAGGAATTCCTCAGGG 1080

HEN3 AAGTCACCAAGAAGGTTATAACTGAGAACATTGCTCAACCAACTAAGGAATTCCTCAGGG 1080

********************* **** ********************** ***** * **

FJ2 AGGTTCCTCATACTACCATGAAAGTCTTGAATGAAAACATTGCTCAACCTGCCAAGGAAA 1140

GS AGGTTCCTCATACTACCATGAAAGTCTTGAATGAAAACATTGCTCAACCTGCCAAGGAAA 1140

FJ1 AGGTTCCTCATACTGCCATGAAAGTCTTGAATGAAAACATTGCTCAACCTGCCAAGGAAA 1140

GX AGGTTCCTCATACTACCATGAAAGTCTTGAATGAAAACATTGCTCAACCTGCCAAGGAAA 1140

HAN AGGTTCCTCATACTACCATGAAAGTCTTGAATGAAAACATTGCTCAACCTGCCAAGGAAA 1140

YN1 AGGTTCCTCATACTACCATGAAAGTCTTGAATGAAAACATTGCTCAACCTGCCAAGGAAA 1140

YN2 AGGTTCCTCATACTACCATGAAAGTCTTGAATGAAAACATTGCTCAACCTGCCAAGGAAA 1140

CQ AGGTTCCTCATACTACCATGAAAGTCTTGAGTGAAAACATTGCTCAACCTGCCAAGGAAA 1140

HEN2 AGGTTCCTCATACTACCATGAAAGTCTTGAATGAAAACATTGCTCAACCTGCCAAGGAAA 1140

HEN1 AGGTTCCTCATGCTACCATGAAAGTCTTGAATGAAAACATTGCTCAACCTGCCAAGGAAA 1140

HEN3 AGGTTCCTCATGCTACCATGAAAGTCTTGAATGAAAACATTGCTCAACCTGCCAAGGAAA 1140

*********** ** *************** *****************************

FJ2 TCATACATGAGTTTGGTACTGGCGCCAAGAATTTCATTTCCGCAGCCCATGAAGGTACTA 1200

GS TCATACATGAGTTTGGTACTGGCGCCAAGAATTTCATTTCCGCAGCCCATGAAGGTACTA 1200

FJ1 TCATACATGAGTTTGGTACTGGCGCCAAGAATTTCATTTCCGCAGCCCATGAAGGTACTA 1200

GX TCATACATGAGTTTGGTACTGGCGCCAAGAATTTCATTTCCGCAGCCCATGAAGGTACTA 1200

HAN TCATACATGAGTTTGGTACTGGCGCCAAGAATTTCATTTCCGCAGCCCATGAAGGTACTA 1200

YN1 TCATACATGAGTTTGGTACTGGCGCCAAGAATTTCATTTCCGCAGCCCATGAAGGTACTA 1200

YN2 TCATACATGAGTTTGGTACTGGCGCCAAGAATTTCATTTCCGCAGCCCATGAAGGTACTA 1200

CQ TCATACATGAGTTTGGTACTGGCGCCAAGAATTTCATTTCCGCAGCCCATGAAGGTACTA 1200

HEN2 TCATACATGAGTTTGGTACTGGCGCCAAGAATTTCATTTCCGCAGCCCATGAAGGTACTA 1200

HEN1 TCATACATGAGTTTGGTAGTGGTGCTAAGAATTTCATTACTTCAGCCCACCAAGGTACCA 1200

HEN3 TCATACATGAGTTTGGTAGTGGTGCTAAGAATTTCATTACTTCAGCCCACCAAGGTACCA 1200

****************** *** ** ************ * ******* ******* *

FJ2 AGCAGTTCTTAAACGAAACTGTTGGCCAACCTACAAAGGAATTCCTGAACGGAGCTCTAG 1260

GS AGCAGTTCTTAAACGAAACTGTTGGCCAACCTACAAAGGAATTCCTGAACGGAGCTCTAG 1260

FJ1 AGCAGTTCTTAAACGAAACTGTTGGCCAACCTACAAAGGAATTCCTGAACGGAGCTCTAG 1260

GX AGCAGTTCTTAAACGAAACTGTTGGCCAACCTACAAAGGAATTCCTGAACGGAGCTCTAG 1260

HAN AGCAGTTCTTAAACGAAACTGTTGGCCAACCTACAAAGGAATTCCTGAACGGAGCTCTAG 1260

YN1 AGCAGTTCTTAAACGAAACTGTTGGCCAACCTACAAAGGAATTCCTGAACGGAGCTCTAG 1260

YN2 AGCAGTTCTTAAACGAAACTGTTGGCCAACCTACAAAGGAATTCCTGAACGGAGCTCTAG 1260

CQ AGCAGTTCTTAAACGAAACTGTTGGCCAACCTACAAAGGAATTCCTGAACGGAGCTCTAG 1260

HEN2 AGCAGTTCTTAAACGAAACTGTTGGCCAACCTACAAAGGAATTCCTGAACGGAGCTCTAG 1260

HEN1 AGCAGTTCTTGGACGAGAACATTGGTCAACCTGCTAAGGAGTTCCTCAGTGGAGCTTTAG 1260

HEN3 AGCAGTTCTTGGACGAGAACATTGGTCAACCTGCTAAGGAGTTCCTCAATGGAGCTTTAG 1260

********** **** * **** ****** * ***** ***** * ****** ***

FJ2 AAACTACTAAAGACGCATTACACCATCTGGGTAAATCATCAGACGAAGCCAACACTTATG 1320

GS AAACTACTAAAGACGCATTACACCATCTGGGTAAATCATCAGACGAAGCCAACATTTATG 1320

FJ1 AAACTACTAAAGACGCATTACACCATCTGGGTAAATCATCAGACGAAGCCAACATTTATG 1320

GX AAACTACTAAAGACGCATTACACCATCTGGGTAAATCATCAGACGAAGCCAACATTTATG 1320

HAN AAACTACTAAAGACGCATTACACCATCTGGGTAAATCATCAGACGAAGCCAACATTTATG 1320

YN1 AAACTACTAAAGACGCATTACACCATCTGGGTAAATCATCAGACGAAGCCAACATTTATG 1320

YN2 AAACTACTAAAGACGCATTACACCATCTGGGTAAATCATCAGACGAAGCCAACATTTATG 1320

CQ AAACTACTAAAGACGCATTACACCATCTGGGTAAATCATCAGACGAAGCCAACATTTATG 1320

HEN2 AAACTACTAAAGACGCATTACACCATCTGGGTAAATCATCAGACGAAGCCAACATTTATG 1320

HEN1 AAACTACTAAGGATGCATTGCACCATCTAGGAAAATCATCTGAGGAATCCAACATTTATG 1320

HEN3 AAACTACTAAGGATGCATTGCACCATCTAGGAAAATCATCTGAGGAATCCAACATTTATG 1320

********** ** ***** ******** ** ******** ** *** ****** *****

FJ2 ATGCCTCGGAAAATACCACTCAGTCTAACGACTCAACT 1358

GS ATGCCTCGGAAAATACCACTCAGTCTAACGACTCAACT 1358

FJ1 ATGCCTCGGAAAATACCACTCAGTCTAACGACTCAACT 1358

GX ATGCCTCGGAAAATACCACTCAGTCTAACGACTCAACT 1358

HAN ATGCCTCGGAAAATACCACTCAGTCTAACGACTCAACT 1358

YN1 ATGCCTCGGAAAATACCACTCAGTCTAACGACTCAACT 1358

YN2 ATGCCTCGGAAAATACCACTCAGTCTAACGACTCAACT 1358

CQ ATGCCTCGGAAAATACCACTCAGTCTAACGACTCAACT 1358

HEN2 ATGCCTCGGAAAATACCACTCAGTCTAACGACTCAACT 1358

HEN1 ATGCCACGGAAAATACCACTCAGTCTAACGACTCAACT 1358

HEN3 ATGCCACGGAAAATACCACTCAGTCTAACGACTCAACT 1358

***** ********************************

**Supplementary Figure 1a.** **Alignment of partial nucleotide sequences of *rap-1a* gene from different Chinese isolates.** FJ2: *B. bovis rap-1a* isolate Fujian 2; GS: *B. bovis rap-1a* isolate Guansu; FJ1: *B. bovis rap-1a* isolate Fujian 1;GX: *B. bovis rap-1a* isolate Guangxi; HAN: *B. bovis rap-1a* isolate Hainan; YN1: *B. bovis rap-1a* isolate Yunnan 1; YN2: *B. bovis rap-1a* isolate Yunnan 2; CQ: *B. bovis rap-1a* isolate Chongqing; HEN2: *B. bovis rap-1a* isolate Henan 2; HEN 1: *B. bovis rap-1a* isolate Henan 1; HEN3: *B. bovis rap-1a* isolate Henan3. Nucleotide substitutions indicated with yellow background. Among these substitutions, nucleotide substitutions affected amino acid modifications indicated with red background.


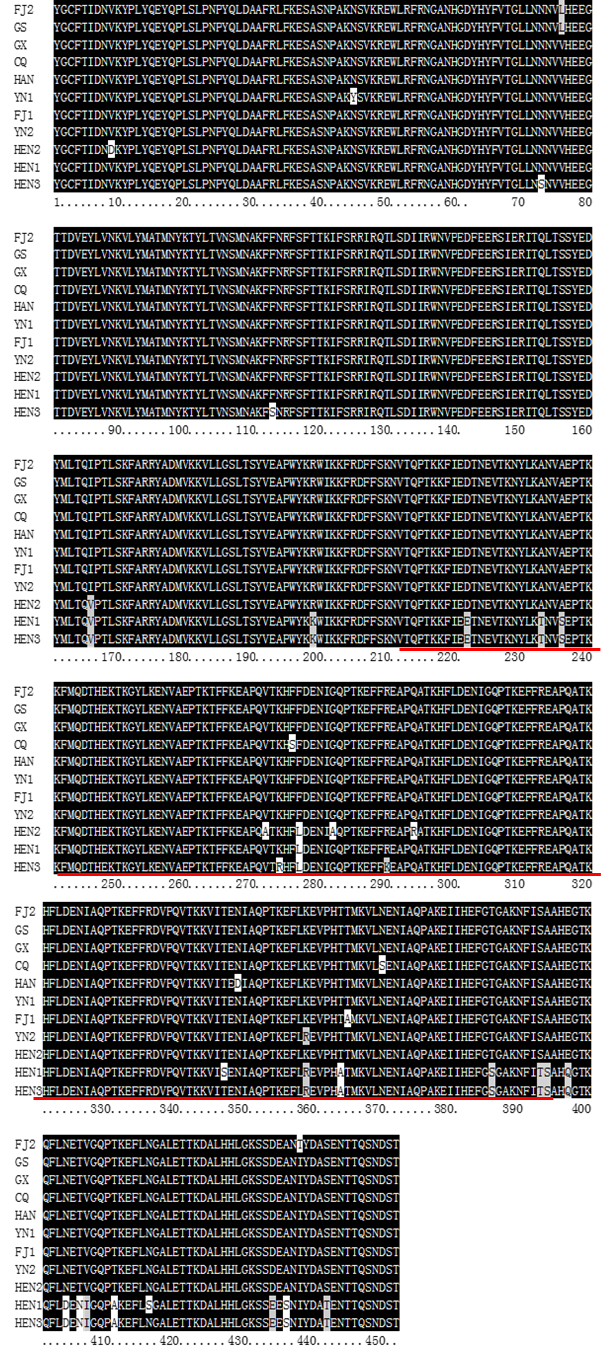


**Supplementary Figure 1b. Alignment of partial amino acid sequences of rap-1a gene from different Chinese isolates.** FJ2: *B. bovis* RAP-1a isolate Fujian 2; GS: *B. bovis* RAP-1a isolate Guansu; GX: *B. bovis* RAP-1a isolate Guangxi; CQ: *B. bovis* RAP-1a isolate Chongqing; HAN: *B. bovis* RAP-1a isolate Hainan; YN1: *B. bovis* RAP-1a isolate Yunnan 1; FJ1: *B. bovis* RAP-1a isolate Fujian 1; YN2: *B. bovis* RAP-1a isolate Yunnan 2; HEN2: *B. bovis* RAP-1a isolate Henan 2; HEN 1: *B. bovis* RAP-1a isolate Henan 1; HEN3: *B. bovis* RAP-1a isolate Henan3. Repeats in RAP-1a gene from different isolates are underlined.
