# Supplementary material for: Genetic characterization and molecular survey of Babesia bovis, Babesia bigemina and Babesia ovata in cattle, dairy cattle and yaks in China
Source: Parasit Vectors. 2015 Oct 9;8:518. doi: 10.1186/s13071-015-1110-0 (PMC4600270; doi:10.1186/s13071-015-1110-0)
Supplement: Additional file 2: — Variability of B. bigemina rap-1c gene. Positions that differ between the different isolates of sequences obtained from gene amplification and cloning for each isolate. (DOC 46 kb) [file 13071_2015_1110_MOESM2_ESM.doc]

Supplementary table 1 Variability of *B. bigemina rap-1c* gene. Positions that differ between the different isolates of sequences obtained from gene amplification and cloning for each isolate.

| **Isolate name** | **variants** | **Variable nucleotide positions and compositions** | | | | | | | | | | | | | | | |
| --- | --- | --- | --- | --- | --- | --- | --- | --- | --- | --- | --- | --- | --- | --- | --- | --- | --- |
|  |  | 88 | 160 | 364 | 391 | 454 | 463 | 481 | 602 | 608 | 610 | 664 | 749 | 751 | 889 | 969 | 1024 |
| CQ | C-1 |  |  |  |  |  |  |  |  |  |  |  |  |  | **T** |  |  |
| GX | G-1 |  |  |  |  |  |  |  |  |  |  |  |  |  |  |  |  |
|  | G-2 | **T** |  |  |  |  |  |  |  |  |  |  |  |  |  |  |  |
| FJ | F-1 |  |  |  |  |  |  |  |  |  | **G** | **C** |  |  |  |  |  |
|  | F-2 |  |  |  |  |  | **T** |  |  |  |  |  |  |  |  |  |  |
| HB | HB-1 |  |  |  |  |  |  |  |  |  |  |  |  |  |  |  |  |
| HAN | HAN-1 |  |  |  | **T** |  |  |  |  |  |  |  |  |  |  |  |  |
|  | HAN-2 |  |  |  |  |  |  | **G** |  |  |  |  |  |  |  | **C** |  |
| YN | Y-1 |  | **C** | **C** |  | **T** |  |  | **A** | **C** |  |  | **A** | **A** |  |  | **C** |
|  | Y-2 |  | **C** | **C** |  | **T** |  |  | **A** | **C** |  |  | **A** | **A** |  |  | **C** |

Note: Amino acid modifications affected by nucleotide substitutions indicated with under line.
